# Representational interactions during audiovisual speech entrainment: Redundancy in left posterior superior temporal gyrus and synergy in left motor cortex

**DOI:** 10.1371/journal.pbio.2006558

**Published:** 2018-08-06

**Authors:** Hyojin Park, Robin A. A. Ince, Philippe G. Schyns, Gregor Thut, Joachim Gross

**Affiliations:** 1 School of Psychology, Centre for Human Brain Health (CHBH), University of Birmingham, Birmingham, United Kingdom; 2 Institute of Neuroscience and Psychology, University of Glasgow, Glasgow, United Kingdom; 3 Institute for Biomagnetism and Biosignalanalysis, University of Muenster, Muenster, Germany; University College London, United Kingdom of Great Britain and Northern Ireland

## Abstract

Integration of multimodal sensory information is fundamental to many aspects of human behavior, but the neural mechanisms underlying these processes remain mysterious. For example, during face-to-face communication, we know that the brain integrates dynamic auditory and visual inputs, but we do not yet understand where and how such integration mechanisms support speech comprehension. Here, we quantify representational interactions between dynamic audio and visual speech signals and show that different brain regions exhibit different types of representational interaction. With a novel information theoretic measure, we found that theta (3–7 Hz) oscillations in the posterior superior temporal gyrus/sulcus (pSTG/S) represent auditory and visual inputs redundantly (i.e., represent common features of the two), whereas the same oscillations in left motor and inferior temporal cortex represent the inputs synergistically (i.e., the instantaneous relationship between audio and visual inputs is also represented). Importantly, redundant coding in the left pSTG/S and synergistic coding in the left motor cortex predict behavior—i.e., speech comprehension performance. Our findings therefore demonstrate that processes classically described as integration can have different statistical properties and may reflect distinct mechanisms that occur in different brain regions to support audiovisual speech comprehension.

## Author summary

Combining different sources of information is fundamental to many aspects of behavior, from our ability to pick up a ringing mobile phone to communicating with a friend in a busy environment. Here, we have studied the integration of auditory and visual speech information. Our work demonstrates that integration relies upon two different representational interactions. One system conveys redundant information by representing information that is common to both auditory and visual modalities. The other system, which is supported by a different brain area, represents synergistic information by conveying greater information than the linear summation of individual auditory and visual information. Further, we show that these mechanisms are related to behavioral performance. This novel insight opens new ways to enhance our understanding of the mechanisms underlying multi-modal information integration, a fundamental aspect of brain function. These fresh insights have been achieved by applying to brain imaging data a recently developed methodology called the partial information decomposition. This methodology also provides a novel and principled way to quantify the interactions between representations of multiple stimulus features in the brain.

## Introduction

While engaged in a conversation, we effortlessly integrate auditory and visual speech information into a unified perception. Such integration of multisensory information is a key aspect of audiovisual speech processing that has been extensively studied [1–4]. Studies of multisensory integration have demonstrated that, in face-to-face conversation, especially in adverse conditions, observing lip movements of the speaker can improve speech comprehension [4–7]. In fact, some people’s ability to perform lip reading demonstrates that lip movements during speech contain considerable information to understand speech without corresponding auditory information [1, 8], even though auditory information is essential to understand speech accurately [9].

Turning to the brain, we know that specific regions are involved in audiovisual integration. Specifically, the superior temporal gyrus/sulcus (STG/S) responds to integration of auditory and visual stimuli, and its disruption leads to reduced McGurk fusion [10–14]. However, these classic studies present two shortcomings. First, their experimental designs typically contrasted two conditions: unisensory (i.e., audio or visual cues) and multisensory (congruent or incongruent audio and visual cues). However, such contrast does not dissociate effects of integration per se from those arising from differences in stimulation complexity (i.e., one or two sources) that could modulate attention, cognitive load, and even arousal. A second shortcoming is that previous studies typically investigated (changes of) regional activation and not information integration between audiovisual stimuli and brain signals. Here, we address these two shortcomings and study the specific mechanisms of audiovisual integration from brain oscillations. We used a novel methodology (speech-brain entrainment) and novel information theoretic measures (the partial information decomposition [PID] [15]) to quantify the interactions between audiovisual stimuli and dynamic brain signals.

Our methodology of speech-brain entrainment builds on recent studies suggesting that rhythmic components in brain activity that are temporally aligned to salient features in speech—most notably the syllable rate [5, 6, 16–18]—facilitate processing of both the auditory and visual speech inputs. The main advantage of speech-brain entrainment is that it replaces unspecific measures of activation with measures that directly quantify the coupling between the components of continuous speech (e.g., syllable rate) and frequency-specific brain activity, thereby tapping more directly into the brain mechanisms of speech segmentation and coding [17].

**Fig 1:**
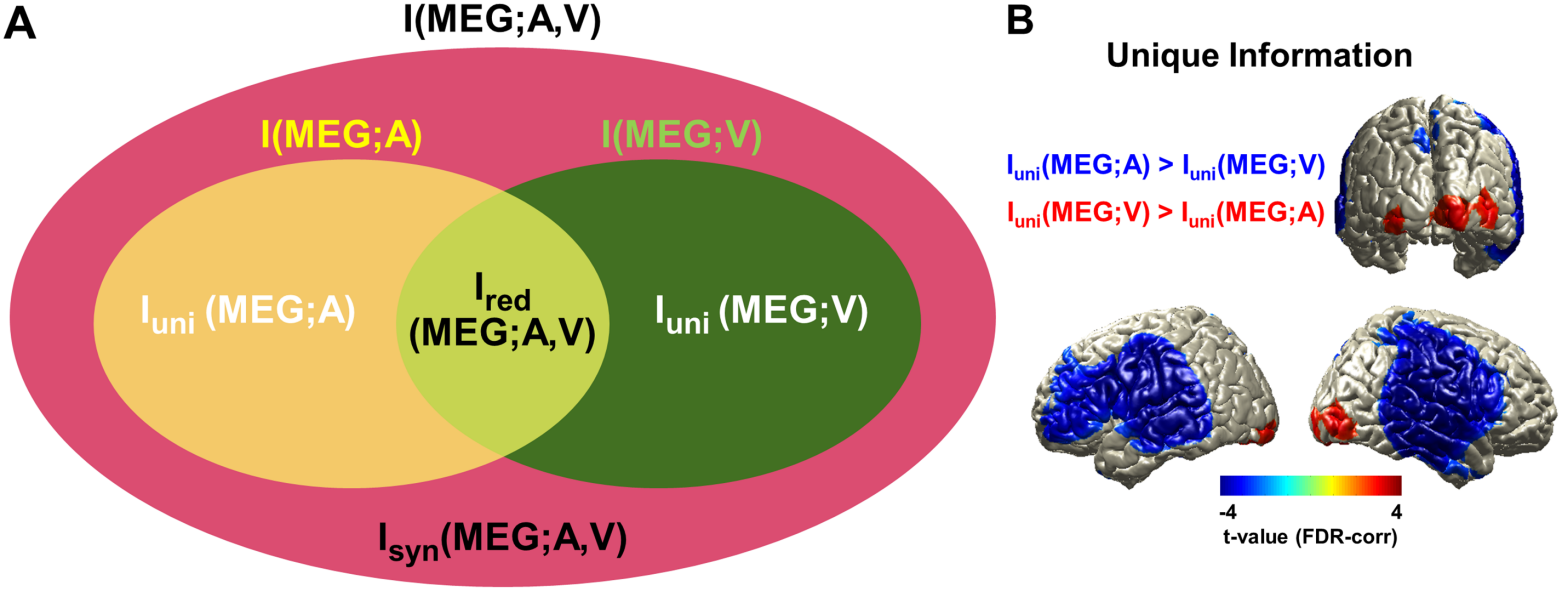
PID of audiovisual speech processing in the brain. (A) Information structure of multisensory audio and visual inputs (sound envelope and lip movement signal) predicting brain response (MEG signal). Ellipses indicate total mutual information I(MEG;A,V), mutual information I(MEG;A), and mutual information I(MEG;V); and the four distinct regions indicate unique information of auditory speech I_uni_(MEG;A), unique information of visual speech I_uni_(MEG;V), redundancy I_red_(MEG;A,V), and synergy I_syn_(MEG;A,V). See Materials and methods for details. See also Ince [15], Barrett [21], and Wibral and colleagues [22] for general aspects of the PID analysis. (B) Unique information of visual speech and auditory speech was compared to determine the dominant modality in different areas (see S1 Fig for more details). Stronger unique information for auditory speech was found in bilateral auditory, temporal, and inferior frontal areas, and stronger unique information for visual speech was found in bilateral visual cortex (*P* < 0.05, FDR corrected). The underlying data for this figure are available from the Open Science Framework (https://osf.io/hpcj8/). *Figure modified from [15, 21, 22] to illustrate the relationship between stimuli in the present study.* FDR, false discovery rate; MEG, magnetoencephalography; PID, partial information decomposition.

In the present study, we used a recently developed information theoretic framework called PID (see Fig 1A and Materials and methods for details) [15, 19, 20]. We consider a three-variable system with a target variable M (here magnetoencephalography [MEG]) and two predictor variables A and V (here audio and visual speech signals), with both A and V conveying information about the target M. Conceptually, the redundancy is related to whether the information conveyed by A and V is the same or different. If the variables are fully redundant, then this means either alone is enough to convey all the information about M (i.e., obtain an optimal prediction of M), and adding observation of the second modality has no benefit for predicting the MEG signal M. The concept of synergy is related to whether A and V convey more information when observed simultaneously, so the prediction of M is enhanced by simultaneous observation of the values of A and V [15]. This means M also represents the instantaneous relationship between A and V. For example, if M is given by the difference between A and V at each sample, then observing either A or V alone tells little about the value of M, but observing them together completely determines it. The PID provides a methodology to rigorously quantify both redundancy and synergy, as well as the unique information in each modality. Unique information is the prediction of the MEG that can be obtained from observing A alone but that is not redundantly available from observing V.

The PID framework therefore addresses a perennial question in multisensory processing: the extent to which each sensory modality contributes uniquely to sensory representation in the brain versus how the representation of different modalities interact (e.g., audio and visual). The PID provides a principled approach to investigate different cross-modal representational interactions (redundant and synergistic) in the human brain during naturalistic audiovisual speech processing—that is, to understand how neural representations of dynamic auditory and visual speech signals interact in the brain to form a unified perception.

Specifically, we recorded brain activity using MEG while participants attended to continuous audiovisual speech to entrain brain activity. We applied the PID to reveal where and how speech-entrained brain activity in different regions reflects different types of auditory and visual integration. In the first experimental condition, we used naturalistic audiovisual speech for which attention to visual speech was not critical (“All congruent” condition). In the second condition, we added a second interfering auditory stimulus that was incongruent to the congruent audiovisual stimuli (“AV congruent” condition), requiring attention to visual speech to suppress the competing additional incongruent auditory input. In the third condition, both auditory stimuli were not congruent to visual stimulus (“All incongruent”). This allows us to see how the congruence of audiovisual stimuli changes integration. We contrasted measures of redundant and synergistic cross-modal interactions between the conditions to reveal differential effects of attention and congruence on multisensory integration mechanisms and behavioral performance.

## Results

We first studied PID in an “All congruent” condition (diotic presentation of speech with matching video) to understand multisensory representational interactions in the brain during processing of natural audiovisual speech. We used mutual information (MI) to quantify the overall dependence between the full multisensory dynamic stimulus time course (broadband speech amplitude envelope and lip area for auditory and visual modalities, respectively) and the recorded brain activity. To determine the dominant modality in each brain area, we statistically compared the auditory unique information to visual unique information across subjects. Note that here, auditory unique information is unique in the context of our PID analysis. Specifically, it quantifies information about the MEG response, which is available only from the auditory speech envelope and not from the visual lip area. The same is true for unique visual information. The analysis revealed stronger visual entrainment in bilateral visual cortex and stronger auditory entrainment in bilateral auditory, temporal, and inferior frontal areas (paired two-sided *t* test, df: 43, *P* < 0.05, false discovery rate [FDR] corrected; Fig 1B).

**Fig 2:**
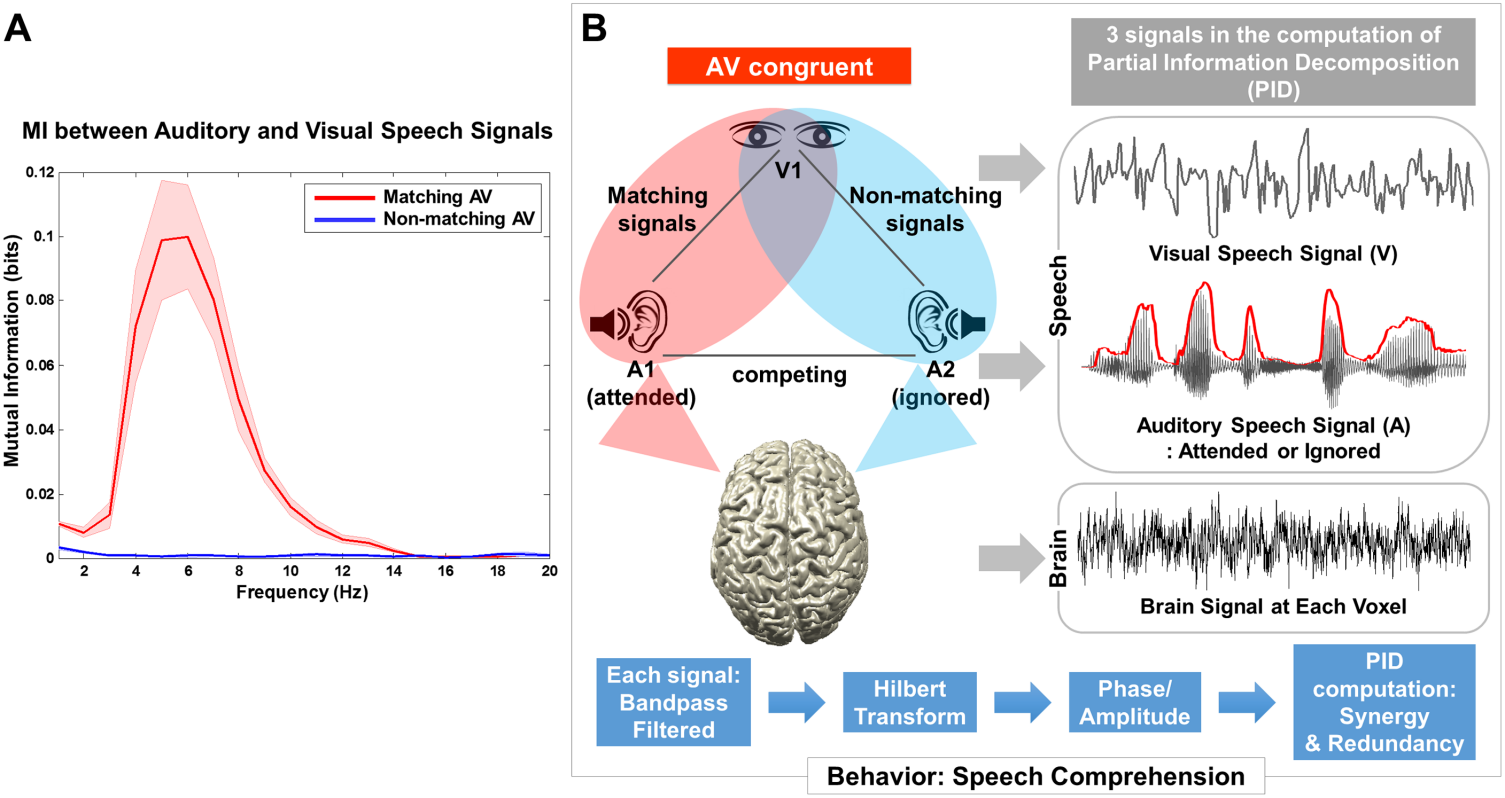
MI between auditory and visual speech signals. (A) To investigate PID in “AV congruent” condition, first MI between auditory speech and visual speech signals was computed separately for matching and nonmatching signals. MI for matching auditory-visual speech signals shows a peak around 5 Hz (red line), whereas MI for nonmatching signals is flat (blue line). The underlying data for this figure are available from the Open Science Framework (https://osf.io/hpcj8/). (B) Analysis of PID is shown for “AV congruent” condition in which both matching and nonmatching auditory-visual speech signals are present on the same brain response (MEG data). Two external speech signals (auditory speech envelope and lip movement signal) and brain signals were used in the PID computation. Each signal was band-pass filtered, followed by Hilbert transform. MEG, magnetoencephalography; MI, mutual information; PID, partial information decomposition.

**Fig 3:**
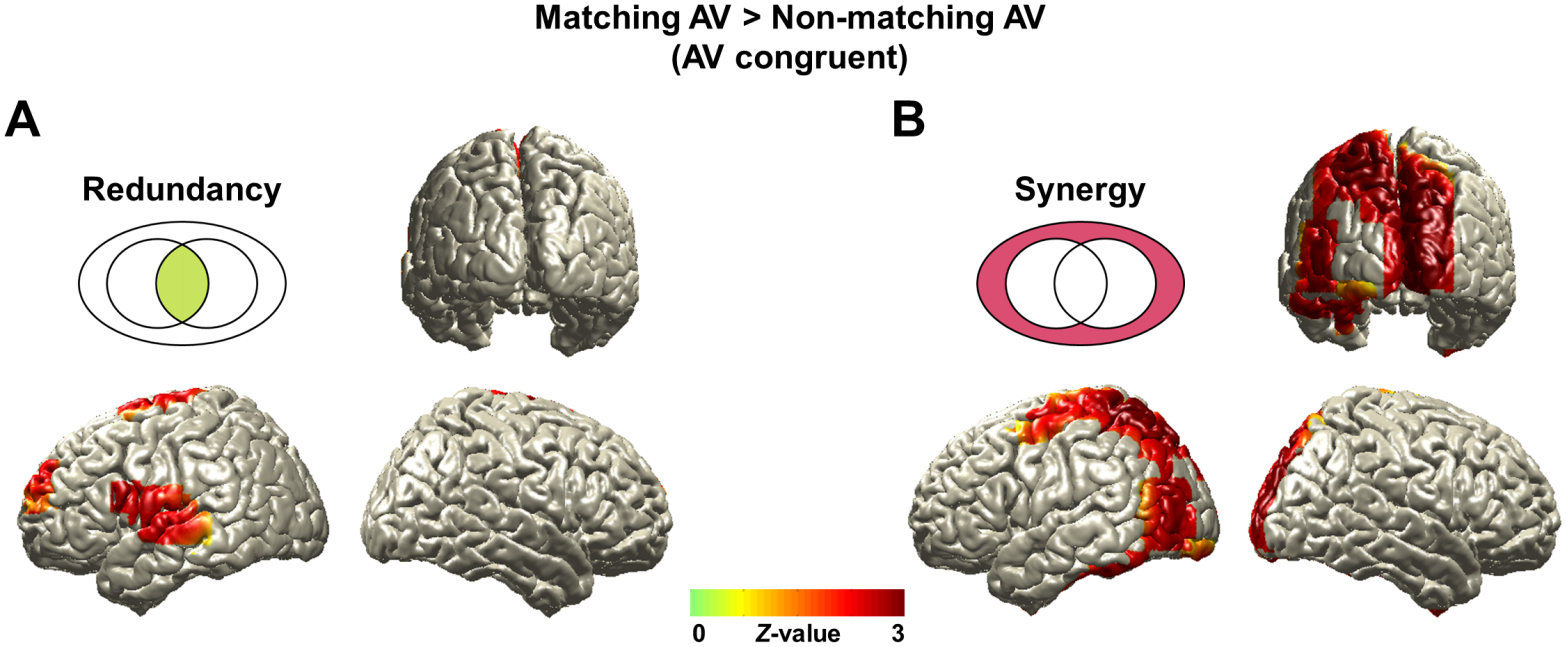
Redundancy and synergy revealed by PID for attention-modulated speech processing (“AV congruent” condition). Redundant and synergistic information of matching audiovisual speech signals in the brain compared to nonmatching signals are shown. Each map (matching or nonmatching in each information map) was firstly yielded to regression analysis using speech comprehension and then transformed to standard Z maps and subtracted. (A) Redundant information is localized in left auditory and superior and middle temporal cortices. (B) Synergistic information is found in left motor and bilateral visual areas (*Z*-difference map at *P* < 0.005). The underlying data for this figure are available from the Open Science Framework (https://osf.io/hpcj8/). PID, partial information decomposition.

**Fig 4:**
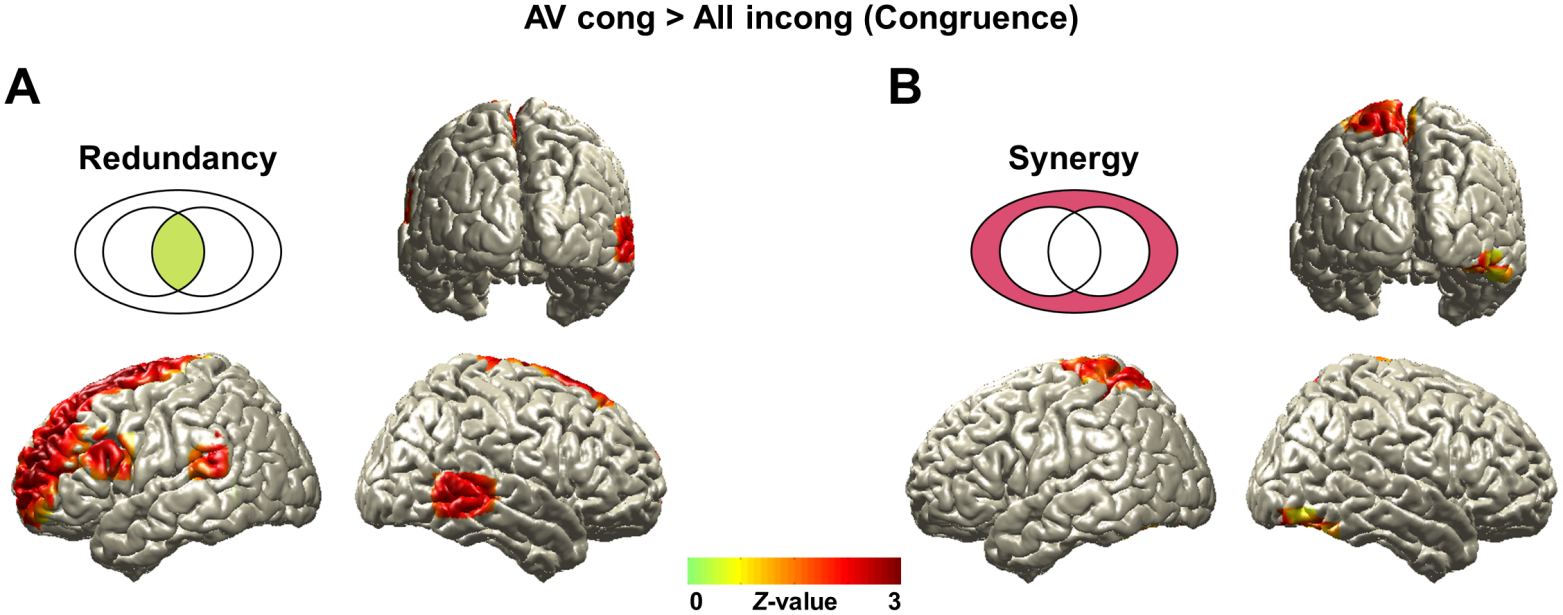
Redundancy and synergy in congruence effect. Comparison between conditions of matching versus nonmatching audiovisual speech signals in “AV congruent” condition entails both attention and congruence effects. To separate this effect, we additionally analyzed contrast for congruence (“AV congruent” > “All incongruent”) first. (A) Redundancy for congruence effect is observed in left inferior frontal region and pSTG/S and right posterior middle temporal cortex (*Z*-difference map at *P* < 0.005). (B) Synergistic information for congruence effect is found in superior part of somatosensory and parietal cortices in left hemisphere (*Z*-difference map at *P* < 0.005). The underlying data for this figure are available from the Open Science Framework (https://osf.io/hpcj8/). pSTG/S, posterior superior temporal gyrus/sulcus.

**Fig 5:**
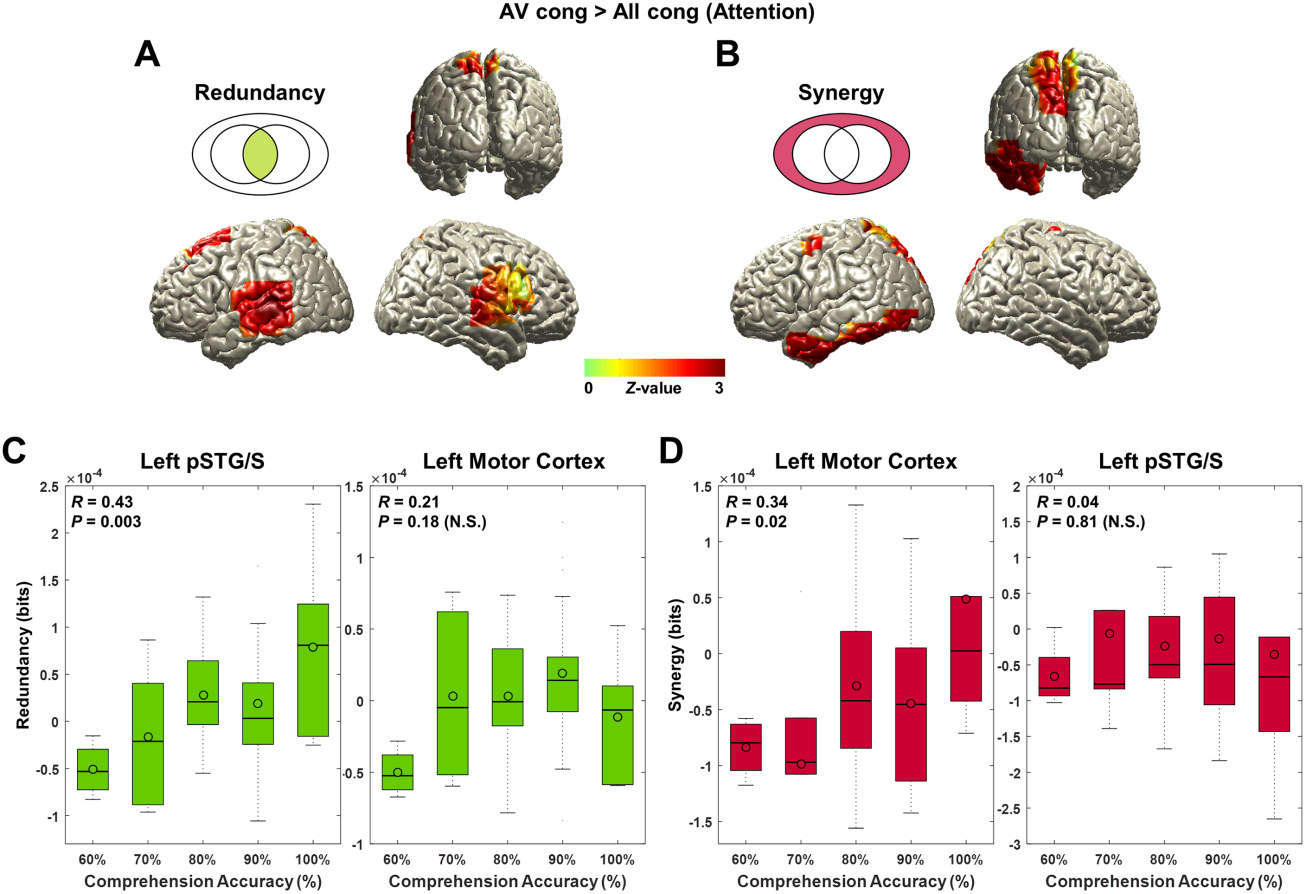
Redundancy and synergy in attention effect. Redundancy and synergy in attention (“AV congruent” > “All congruent”) are analyzed. Further, to explore whether this effect is specific to “AV congruent” condition (not because of decreased information in “All congruent” condition), we extracted raw values of each information map at the local maximum voxel and correlated it with speech comprehension accuracy across subjects. (A) Redundancy for attention effect was observed in left auditory and temporal (superior and middle temporal cortices and pSTG/S) areas and right inferior frontal and superior temporal cortex (*Z*-difference map at *P* < 0.005). (B) Synergistic information for attention effect was localized in left motor cortex, inferior temporal cortex, and parieto-occipital areas (*Z*-difference map at *P* < 0.005). (C) Redundancy at the left posterior superior temporal region in “AV congruent” condition was found to be positively correlated with speech comprehension accuracy (*R* = 0.43, *P* = 0.003). However, this redundant representation was not found for left motor cortex where synergistic information was represented (*R* = 0.21, *P* = 0.18). (D) Synergy at the left motor cortex in “AV congruent” condition was also positively correlated with speech comprehension accuracy across subjects (*R* = 0.34, *P* = 0.02). Likewise, synergistic representation was not found to be related to comprehension in the left posterior superior temporal region where redundant information was represented (*R* = 0.04, *P* = 0.81). This finding suggests that redundant information in the left posterior superior temporal region and synergistic information in the left motor cortex in a challenging audiovisual speech condition support better speech comprehension. The underlying data for this figure are available from the Open Science Framework (https://osf.io/hpcj8/). N.S., not significant; pSTG/S, posterior superior temporal gyrus/sulcus.

To identify a frequency band at which auditory and visual speech signals show significant dependencies, we computed MI between both signals and compared it to MI between nonmatching auditory and visual speech signals for frequencies from 0 to 20 Hz. Here, we used all the talks in the present study to delineate the spectral profile of dependencies between matching or nonmatching auditory and visual speech signals. As expected, only matching audiovisual speech signals show significant MI peaking at 5 Hz (Fig 2A), consistent with previous results based on coherence measure (see Fig 2C in [5]). Based on this, we focus our further analysis on the 3–7 Hz frequency band (5 ± 2 Hz) in the following analyses (Figs 3–5). This frequency range is known to correspond to the syllable rate in continuous speech [16] and within which amplitude envelope of speech is known to reliably entrain auditory brain activity [18–23].

### Redundancy in left posterior superior temporal gyrus/sulcus (pSTG/S) and synergy in left motor cortex

Next, we investigated how multimodal representational interactions are modulated by attention and congruence in continuous audiovisual speech. Here, we focus on an “AV congruent” condition in which a congruent audiovisual stimulus pair is presented monaurally together with an interfering nonmatching auditory speech stimulus to the other ear (Fig 2B). This condition is of particular interest because visual speech (lip movement) is used to disambiguate the two competing auditory speech signals. Furthermore, it is ideally suited for our analysis because we can directly contrast representational interactions quantified with the PID in matching and nonmatching audiovisual speech signals in the same data set (see Fig 2B).

Fig 3 shows corrected group statistics for the contrast of matching and nonmatching audiovisual speech in the “AV congruent” condition. Redundant information is significantly stronger in left auditory and superior and middle temporal cortices (Fig 3A; Z-difference map at *P* < 0.005) for matching compared to nonmatching audiovisual speech. In contrast, significantly higher synergistic information for matching compared to nonmatching audiovisual speech is found in left motor and bilateral visual areas spreading along dorsal and ventral stream regions of speech processing [24] (Fig 3B; Z-difference map at *P* < 0.005). Next, we tested attention and congruence effects separately because the contrast of matching versus nonmatching audiovisual speech confounds both effects.

First, the congruence effect (“AV congruent” > “All incongruent”) shows higher redundant information in left inferior frontal region (BA 44/45) and posterior superior temporal gyrus and right posterior middle temporal cortex (Fig 4A; Z-difference map at *P* < 0.005) and higher synergistic information in superior part of somatosensory and parietal cortices in left hemisphere (Fig 4B; Z-difference map at *P* < 0.005).

The attention effect (“AV congruent” > “All congruent”) shows higher redundant information in left auditory and temporal (superior, middle, and inferior temporal cortices and pSTG/S) areas and right inferior frontal and superior temporal cortex (Fig 5A; *Z*-difference map at *P* < 0.005). Higher synergistic information was localized in left motor cortex, inferior temporal cortex, and parieto-occipital areas (Fig 5B; *Z*-difference map at *P* < 0.005).

In summary, theta-band activity in left pSTG/S represents redundant information about audiovisual speech significantly more strongly in experimental conditions with higher attention and congruence. In contrast, synergistic information in the left motor cortex is more prominent in conditions requiring increased attention. Therefore, the increased relevance of visual speech in the “AV congruent” condition leads to increased redundancy in left pSTG/S and increased synergy in left motor cortex. This differential effect on representational interactions may reflect different integration mechanisms operating in the different areas. For detailed local maps of interaction between predictors (auditory and visual speech signals) and target (MEG response), see S3 Fig.

### Performance scales with redundancy in left pSTG/S and synergy in left motor cortex

Next, we investigated if the differential pattern of redundancy and synergy is of behavioral relevance in our most important condition—"AV congruent"—in which visual speech is particularly informative. To this end, we extracted raw values of redundancy for the location showing strongest redundancy in the left pSTG/S in Fig 5A and synergy for the location showing strongest synergy in the left motor cortex in Fig 5B for “AV congruent” condition. After normalization with surrogate data (see Materials and methods section), we computed correlation with performance measures (comprehension accuracy) across participants. Both redundancy in left pSTG/S (*R* = 0.43, *P* = 0.003; Fig 5C) and synergy in left motor cortex (*R* = 0.34, *P* = 0.02; Fig 5D) are significantly correlated with comprehension accuracy. These results suggest that the redundancy in left pSTG/S and synergy in left motor cortex under challenging conditions (i.e., in the presence of distracting speech) are related to perceptual mechanisms underlying comprehension.

## Discussion

In this study, we investigated how multisensory audiovisual speech rhythms are represented in the brain and how they are integrated for speech comprehension. We propose to study multisensory integration using information theory for the following reasons: First, by directly quantifying dynamic encoded representation of speech stimuli, our results are more clearly relevant to information-processing mechanisms than are differences in activation between blocks of stimulus conditions. Second, cross-modal interactions can be quantified directly within a naturalistic multimodal presentation without requiring contrasts between multimodal and unimodal conditions (e.g., AV > A + V). Third, the PID provides measures of representational interactions that address questions that are not available with other statistical approaches (particularly synergy; Fig 1A) [15, 21, 22].

We found that left posterior superior temporal region represents speech information that is common to both auditory and visual modalities (redundant), while left motor cortex represents information about the instantaneous relationship between audio and visual speech (synergistic). These results are obtained from low-frequency theta rhythm (3–7 Hz) signals corresponding to syllable rate. Importantly, redundancy in pSTG/S and synergy in left motor cortex predict behavioral performance—speech comprehension accuracy—across participants.

A critical hallmark of multisensory integration in general, and audiovisual integration in particular, is the behavioral advantage conveyed by both stimulus modalities as compared to each single modality. Here, we have shown that this process may rely on at least two different mechanisms in two different brain areas, reflected in different representational interaction profiles revealed with information theoretic synergy and redundancy.

### What do redundancy and synergy mean? Linking to audiovisual integration in functional magnetic resonance imaging (fMRI) studies

In fMRI studies, audiovisual speech integration has been studied using experimental conditions that manipulate the stimulus modalities presented (e.g., [13, 25]). Changes in blood oxygen level–dependent (BOLD) responses elicited by congruent audiovisual stimuli (AV) have been compared to auditory-only (AO), visual-only (VO), their sum (AO + VO), or their conjunction (AO ∩ VO). Greater activation for the congruent audiovisual condition (AV) compared to others has been interpreted as a signature of audiovisual speech integration. Comparison to auditory-only (AO) activation or visual-only (VO) activation has been regarded as a less conservative criterion for integration, since even if auditory and visual stimuli caused independent BOLD activity that combined linearly, this contrast would reveal an effect. To address this, comparison to the summation of the unimodal activations (AO + VO) has been used to demonstrate supra-additive activation, which is more suggestive of a cross-modal integration process. Rather than overall activation while the stimulus is present, the information theoretic approach instead focuses on quantifying the degree to which the changing speech time course is encoded or represented in the neural signals. The MI calculated here is an effect size for the ongoing entrainment of the MEG time course by the time varying speech—i.e., it quantifies the strength of the representation of dynamic audiovisual speech in the neural activity. While the basic expression on which our redundancy measure is based (Materials and methods, Eq 1) looks similar to an activation contrast (e.g., sum versus conjunction), it is important to keep in mind that this is about the strength of the dynamic low-frequency entrainment in each modality, not simply overall activation contrasts between conditions as in the classic fMRI approach.

The PID can quantify the representational interactions between multiple sensory signals and the associated brain response in a single experimental condition in which both sensory modalities are simultaneously present. In the PID framework, the unique contributions of a single (e.g., auditory) sensory modality to brain activity are directly quantified when both are present, instead of relying on the statistical contrast between modalities presented independently. Furthermore, the PID method allows the quantification of both redundant and synergistic interactions. In the context of audiovisual integration, both types of interaction can be seen as integration effects. Redundant information refers to quantification of overlapping information content of the predictor variables (auditory and visual speech signals), and synergistic information refers to additional information gained from simultaneous observation of two predictor variables compared to observation of one. Both of these types of interaction quantify multimodal stimulus representation that cannot be uniquely attributed to one of the two modalities. Redundant representation cannot be uniquely attributed, since that part of the brain response could be predicted from either of the stimulus modalities. Synergistic representation cannot be uniquely attributed, since that part of the brain response could only be predicted from simultaneous observation of both modalities and not from either one alone.

Note that these statistical interactions are quite different from interaction terms in a linear regression analysis, which would indicate the (linear) functional relationship between one stimulus modality and the MEG response is modulated by the value of the other stimulus modality. MI is an effect size that can be interpreted, because of its symmetry, from both an encoding and decoding perspective. From an encoding perspective, MI is a measure of how much an observer’s predictive model for possible MEG activity values changes when a specific auditory speech value is observed 100 ms prior. It quantifies the improvement in predictive performance of such an observer when making an optimal guess based on the auditory speech signal they see, over the guess they would make based on overall MEG activity without observing a stimulus value. From this perspective, redundancy quantifies the overlapping or common predictions that would be made by two Bayesian optimal observers, one predicting based on the auditory signal and the other the visual. Synergy is an increase in predictive power when both signals are obtained simultaneously. That is, it is possible to obtain a better prediction of the MEG with simultaneous knowledge of the specific combination of A and V observed than it is from combining only the predictions of the previous two unimodal observers. From considering the local plots (i.e., the values that are summed to obtain the final expectation value) in S3 Fig, we can see that a better prediction of the MEG in left motor cortex is made from the joint multimodal input in the case in which the MEG signal is high (above median), and the auditory and visual signals are in opposite ranges (e.g., high/low or low/high).

Existing techniques like representational similarity analysis (RSA) [26] and cross-decoding [27] can address the same conceptual problem as redundancy but from the angle of similarity of representations on average rather than specific overlapping Bayesian predictive information content within individual samples, which the information theoretic framework provides. Techniques exploiting decoding in different conditions can show the degree to which multimodal representations are similar to unimodal representations [28, 29] and whether there is an improvement in performance when the representation is learned in the multimodal condition. However, PID is explicitly a trivariate analysis considering two explicit quantified stimulus features and the brain signal. The information theoretic definition of synergy means there is enhanced prediction of neural responses from simultaneous multimodal stimuli compared to independent predictions combined from each modality (but still presented together). This differs from typical multimodal versus unimodal contrasts, even those involving decoding, because it explicitly considers the effect of continuous naturalistic variation in both stimulus modalities on the recorded signal.

### Left posterior superior temporal region extracts common features from auditory and visual speech rhythms

Posterior superior temporal region (pSTG/S) has been implicated in audiovisual speech integration area by functional [30–32] and anatomical [33] neuroimaging. A typical finding in fMRI studies is that pSTG/S shows stronger activation for audiovisual (AV) compared to auditory-only (AO) and/or visual-only (VO) conditions. This was confirmed by a combined fMRI-transcranial magnetic stimulation (TMS) study in which the likelihood of McGurk fusion was reduced when TMS was applied individually to fMRI-localized posterior superior temporal sulcus (pSTS), suggesting a critical role of pSTS in auditory-visual integration [14].

The redundant information in the same left superior temporal region in this study matches this notion that this region processes shared information from both modalities. We found this region not only in the congruence effect (“AV congruent” > “All incongruent”; Fig 4A) but also in the attention effect (“AV congruent” > “All congruent”; Fig 5A).

### Left motor cortex activity reflects synergistic information in audiovisual speech processing

We found the left motor cortex shows increased synergy for the matching versus nonmatching audio stimuli of “AV congruent” condition (Fig 3B). However, further analysis optimized for effects of attention and congruence revealed slightly different areas—with the area that shows strongest synergy change with attention (Fig 5B; BA6) located more lateral and anterior compared to the area identified in the congruence (Fig 4B). Previous studies have demonstrated increased phase locking of left motor cortex activity to frequency-tagged stimuli during auditory spatial attention [34, 35]. We extend these findings by demonstrating attention-mediated synergistic interactions of auditory and visual representations in left motor cortex.

The motor region in the attention contrast is consistent with the area in our previous study that showed entrainment to lip movements during continuous speech that correlated with speech comprehension [5]. In another study, we identified this area as the source of top-down modulation of activity in the left auditory cortex [23]. The definition of synergistic information in our context refers to more information gained from the simultaneous observation of auditory and visual speech compared to the observation of each alone. When it comes to the attention effect (“AV congruent” > “All congruent”), “AV congruent” condition requires paying more attention to auditory and visual speech than the “All congruent” condition does, even though the speech signals to be attended match the visual stimulus in both conditions. Thus, this synergy effect in the left motor cortex can be explained by a net attention effect at the same level of stimulus congruence. This effect is likely driven by stronger attention to visual speech, which is informative for the disambiguation of the two competing auditory speech streams [5]. This notion is plausible because it is supported by directional information analysis that shows that the left motor cortex better predicts upcoming visual speech in the “AV congruent” condition, in which attention to visual speech is crucial (S2B and S2D Fig).

However, a number of open questions in need of further investigation still remain. First, the auditory speech envelope and lip area information used in our analysis only capture part of the rich audiovisual information that is available to interlocutors in a real-life conversation. Other, currently unaccounted features might even be correlated across modalities (e.g., a different visual feature that is correlated with the auditory envelope). Since our analysis is restricted to these two features, it is possible that with a richer feature set for each modality, the unique information obtained from each would be reduced. In addition, the auditory speech signal is available at a much higher temporal resolution compared to the lip area signal, leading to a potential bias in the information content of both signals. Since the analysis of speech-brain coupling is a relatively new research field, we envisage methodological developments that will capture more aspects of the rich audiovisual signals. But in the context of our analysis that is focused on syllable components in speech, it seems reasonable to use these two signals that are known to contain clear representations of syllable-related frequencies [18, 36].

Second, it should be noted that we computed PID measures on the speech signals and 100 ms shifted MEG signal as in previous analyses [5, 18, 23] to compensate for delays between stimulus presentation and main cortical responses. We have confirmed that this (on average) maximizes speech-brain coupling. However, different aspects of multisensory integration likely occur at different latencies, especially in higher-order brain areas. This highly interesting but complex question is beyond the scope of the present study but will hopefully be addressed within a similar framework in future studies.

Third, while an unambiguous proof is missing, we believe that converging evidence suggests that participants attended visual speech more in “AV congruent” condition than in the other conditions. Indeed, it seems very unlikely that participants did not attend to visual speech after being explicitly instructed to attend (especially because visual speech provided important task-relevant information in the presence of a distracting auditory input). The converging evidence is based on behavioral performance, eye tracking results, and previous studies. Previous research indicates that the availability of visual speech information improves speech intelligibility under difficult listening conditions [1, 6, 37]. The “AV congruent” condition was clearly more difficult compared to the “All congruent” condition because of the presence of an interfering auditory stimulus. One could argue that participants could accomplish the task by simply using auditory spatial attention. However, our behavioral data (see Fig 1B in [5]) argue against this interpretation. If participants had ignored the visual stimulus and only used auditory spatial attention, then we would expect to see the same behavioral performance between “AV congruent” and “All incongruent” conditions. In both cases, two different auditory stimuli were presented, and only relying on auditory information would lead to the same behavioral performance. Instead, we find a significant difference in behavioral performance between both conditions. The availability of the congruent visual stimulus (in the “AV congruent” condition) resulted in a significant increase of behavioral performance (compared to “All incongruent” condition) to the extent that it reached the performance for the “All congruent” condition (no significant difference between “All congruent” and “AV congruent” conditions measured by comprehension accuracy; mean ± s.e.m; 85.0% ± 1.66% for “All congruent,” 83.40% ± 1.73% for “AV congruent” condition). This is strong evidence that participants actually made use of the visual information. In addition, this is also supported by eye fixation on the speaker’s lip movement, as shown in S5 Fig.

In summary, we demonstrate how information theoretic tools can provide a new perspective on audiovisual integration, by explicitly quantifying both redundant and synergistic cross-modal representational interactions. This reveals two distinct profiles of audiovisual integration that are supported by different brain areas (left motor cortex and left pSTG/S) and are differentially recruited under different listening conditions.

## Materials and methods

### Participants

Data from 44 subjects were analyzed (26 females; age range: 18–30 y; mean age: 20.54 ± 2.58 y). Another analysis of these data was presented in a previous report [5]. All subjects were healthy, right-handed (confirmed by Edinburgh Handedness Inventory [38]), and had normal or corrected-to-normal vision and normal hearing (confirmed by 2 hearing tests using research applications on an iPad: uHear [Unitron Hearing Limited] and Hearing-Check [RNID]). None of the participants had a history of developmental, psychological, or neurological disorders. They all provided informed written consent before the experiment and received monetary compensation for their participation. The study was approved by the local ethics committee (CSE01321; College of Science and Engineering, University of Glasgow) and conducted in accordance with the ethical guidelines in the Declaration of Helsinki.

### Stimuli and experiment

We used audiovisual video clips of a professional male speaker talking continuously (7–9 min), which were used in our previous study [5]. Since in some conditions (“AV congruent,” “All incongruent” conditions) the auditory speeches are delivered dichotically, to ensure that there are no differences other than talks themselves in those conditions, we made all the videos with the same male speaker. The talks were originally taken from TED talks (www.ted.com/talks/) and edited to be appropriate to the stimuli we used (e.g., editing words referring to visual materials, the gender of the speaker, etc.).

High-quality audiovisual video clips were filmed by a professional filming company, with sampling rate of 48 kHz for audio and 25 frames per second (fps) for video in 1,920 × 1,080 pixels.

In order to validate stimuli, 11 videos were rated by 33 participants (19 females; aged 18–31 y; mean age: 22.27 ± 2.64 y) in terms of arousal, familiarity, valence, complexity, significance (informativeness), agreement (persuasiveness), concreteness, self-relatedness, and level of understanding, using Likert scale [39] 1–5 (for an example of concreteness, 1: very abstract, 2: abstract, 3: neither abstract nor concrete, 4: concrete, 5: very concrete). Eight talks were finally selected for the MEG experiment by excluding talks with mean scores of 1 and 5.

Questionnaires for each talk were validated in a separate behavioral study (16 subjects; 13 females; aged 18–23 y; mean age: 19.88 ± 1.71 y). These questionnaires are designed to assess the level of speech comprehension. Each questionnaire consists of 10 questions about a given talk to test general comprehension (e.g., “What is the speaker’s job?”) and were validated in terms of accuracy (the same level of difficulty), response time, and the length (word count).

Experimental conditions used in this study were “All congruent,” “All incongruent,” and “AV congruent.” In each condition (7–9 min), 1 video recording was presented, and 2 (matching or nonmatching) auditory recordings were presented to the left and the right ear, respectively. Half of the 44 participants attended to speech in the left ear, and the other half attended to speech in the right ear.

The “All congruent” condition is a natural audiovisual speech condition in which auditory stimuli to both ears and visual stimuli are congruent (V1A1A1; the first A denotes talk presented to the left ear, and the second A denotes talk presented to the right ear; the number refers to the identity of the talks). The “All incongruent” condition has three different stimulus streams from three different videos, and participants are instructed to attend to auditory information presented to one ear (V1A2A3). The “AV congruent” condition consists of one auditory stimulus matching the visual information, and the speech presented to the other ear serves as a distractor. Participants attend to the talk that matches visual information (V1A1A2 for left ear attention group, V1A2A1 for right ear attention group). Each condition represents one experimental block, and the order of conditions was counterbalanced across subjects.

Participants were instructed to fixate on the speaker’s lip throughout the presentation in all experimental conditions, and we monitored the eye gaze using an eye tracker. Furthermore, we explained the importance of eye fixation on the speaker’s lip movement during the instruction session. They were also informed that for this reason, their eye movement and gaze behavior would be monitored using an eye tracker (see eye tracker data analysis in S5 Fig).

A fixation cross (either yellow or blue color) was overlaid on the speaker’s lip during the whole video for mainly two reasons: (1) to help maintain eye fixation on the speaker’s lip movement and (2) to indicate the auditory stimulus to pay attention to (left or right ear; e.g., “If the color of fixation cross is yellow, please attend to left ear speech”). The color was counterbalanced across subjects (for half of participants, yellow indicates attention to the left ear speech; for another half, attention to the right ear speech). This configuration was kept the same for all experimental conditions to ensure the same video display other than the experimental manipulations we aimed at. However, in “All congruent” condition (natural audiovisual speech), in which 1 auditory stream is presented diotically, attention cannot be directed to left or right ear, so participants were instructed to ignore the color of the fixation cross and just to attend the auditory stimuli naturally. In addition, to prevent stimulus-specific effects, we used 2 sets of stimuli consisting of different combinations of audiovisual talks. These 2 sets were randomized across participants (set 1 for half of participants, set 2 for the other half). For example, talks for “All congruent” condition in set 1 were talks for “AV congruent” condition in set 2.

There was no significant difference in comprehension accuracy between left and right ear attention groups (two-sample *t* test, df: 42, *P* > 0.05). In this study, we pooled across both groups for data analysis so that attentional effects for a particular side (e.g., left or right) are expected to cancel out.

For the recombination and editing of audiovisual talks, we used Final Cut Pro X (Apple, Cupertino, CA). The stimuli were presented with Psychtoolbox [40] in MATLAB (MathWorks, Natick, MA). Visual stimuli were delivered with a resolution of 1,280 × 720 pixels at 25 fps (mp4 format). Auditory stimuli were delivered at a 48 kHz sampling rate via a sound pressure transducer through 2 five-meter-long plastic tubes terminating in plastic insert earpieces.

A comprehension questionnaire was administered about the attended speech separately for each condition.

### Data acquisition

Cortical neuromagnetic signals were recorded using a 248 magnetometers whole-head MEG system (MAGNES 3600 WH, 4-D Neuroimaging) in a magnetically shielded room. The MEG signals were sampled at 1,017 Hz and were denoised with information from the reference sensors using the denoise_pca function in FieldTrip toolbox [41]. Bad sensors were excluded by visual inspection, and electrooculographic (EOG) and electrocardiographic (ECG) artifacts were eliminated using independent component analysis (ICA). An eye tracker (EyeLink 1000, SR Research) was used to examine participants’ eye gaze and movements to ensure that they fixated on the speaker’s lip movements.

Structural T1-weighted MRIs of each participant were acquired at 3 T Siemens Trio Tim scanner (Siemens, Erlangen, Germany) with the following parameters: 1.0 × 1.0 × 1.0 mm^3^ voxels; 192 sagittal slices; field of view (FOV): 256 × 256 matrix.

### Data analysis

Information theoretic quantities were estimated with the Gaussian-Copula Mutual Information (GCMI) method [42] (https://github.com/robince/gcmi). PID analysis was performed with the GCMI approach in combination with an open source PID implementation in MATLAB, which implements the PID [19, 20] with a redundancy measure based on common change in local surprisal [15] (https://github.com/robince/partial-info-decomp). For statistics and visualization, we used the FieldTrip Toolbox [41] and in-house MATLAB codes. We followed the suggested guidelines [43] for MEG studies.

#### MEG-MRI coregistration

Structural MR images of each participant were coregistered to the MEG coordinate system using a semiautomatic procedure. Anatomical landmarks (nasion, bilateral preauricular points) were identified before the MEG recording and also manually identified in the individual’s MR images. Based on these landmarks, both MEG and MRI coordinate systems were initially aligned. Subsequently, numerical optimization was achieved by using the ICP algorithm [44].

#### Source localization

A head model was created for each individual from their structural MRI using normalization and segmentation routines in FieldTrip and SPM8. Leadfield computation was performed based on a single-shell volume conductor model [45] using an 8 mm grid defined on the template provided by MNI (Montreal Neurological Institute). The template grid was linearly transformed into individual head space for spatial normalization. Cross-spectral density matrices were computed using fast Fourier transform on 1 s segments of data after applying multitaper (±2 Hz frequency smoothing [46]). Source localization was performed using DICS beamforming algorithm [47], and beamformer coefficients were computed sequentially for all frequencies from 1 to 20 Hz for the dominant source direction in all voxels with a regularization of 7% of the mean across eigenvalues of the cross-spectral density matrix.

#### Auditory speech signal processing

The amplitude envelope of auditory speech signals was computed following the approach reported in [36]. We constructed 8 frequency bands in the range 100–10,000 Hz to be equidistant on the cochlear map [48]. The auditory sound speech signals were band-pass filtered in these bands using a fourth-order forward and reverse Butterworth filter. Then, Hilbert transform was applied to obtain amplitude envelopes for each band of signal. These signals were then averaged across bands and resulted in a wideband amplitude envelope. For further analysis, signals were downsampled to 250 Hz.

#### Visual speech signal processing

A lip movement signal was computed using an in-house MATLAB script. We first extracted the outline lip contour of the speaker for each frame of the movie stimuli. From the lip contour outline, we computed the frame-by-frame lip area (area within lip contour). This signal was resampled at 250 Hz to match the sampling rate of the preprocessed MEG signal and auditory sound envelope signal. We reported the first demonstration of visual speech entrainment using this lip movement signal [5].

#### Estimating MI and other information theoretic quantities: Shannon’s information theory [49]

Information theory was originally developed to study man-made communication systems; however, it also provides a theoretical framework for practical statistical analysis. It has become popular for the analysis of complex systems in a range of fields and has been successfully applied in neuroscience to spike trains [50, 51], LFPs [52–53], EEG [54, 55], and MEG time series data [18, 23, 56, 57]. MI is a measure of statistical dependence between two variables, with a meaningful effect size measured in bits (see [42] for a review). MI of 1 bit corresponds to a reduction of uncertainty about one variable of a factor 2 after observation of another variable. Here, we estimate MI and other quantities using GCMI [42]. This provides a robust, semiparametric lower bound estimator of MI by combining the statistical theory of copulas with the closed-form solution for the entropy of Gaussian variables. Crucially, this method performs well for higher dimensional responses as required for measuring three-way statistical interactions and allows estimation over circular variables, like phase.

#### MI between auditory and visual speech signals

Following the GCMI method [42], we normalized the complex spectrum by its amplitude to obtain a 2D representation of the phase as points lying on the unit circle. We then rank-normalized the real and imaginary parts of this normalized spectrum separately and used the multivariate GCMI estimator to quantify the dependence between these two 2D signals. This gives a lower bound estimate of the MI between the phases of the two signals.

To determine the frequency of interest for the main analysis (PID), we computed MI between auditory (A) and visual (V) speech signals for the matching AV and nonmatching AV signals from all the stimuli we used. As shown in Fig 2A, there was no relationship between nonmatching auditory and visual stimuli, but there was a frequency-dependent relationship for matching stimuli peaking in the band 3–7 Hz. This is consistent with previous results using coherence measure [5, 36]. This frequency band corresponds to the syllable rate and is known to show robust phase coupling between speech and brain signals.

#### PID theory

We seek to study the relationships between the neural representations of auditory (here amplitude envelope) and visual (here dynamic lip area) stimuli during natural speech. MI can quantify entrainment of the MEG signal by either or both of these stimuli but cannot address the relationship between the two entrained representations—their representational interactions. The existence of significant auditory entrainment revealed with MI demonstrates that an observer who saw a section of auditory stimulus would be able to, on average, make some prediction about the MEG activity recorded after presentation of that stimulus (this is precisely what is quantified by MI). Visual MI reveals the same for the lip area. However, a natural question is then whether these two stimulus modalities provide the same information about the MEG or provide different information. If an observer saw the auditory stimulus and made a corresponding prediction for the MEG activity, would that prediction be improved by observation of the concurrent visual stimulus, or would all the information about the likely MEG response available in the visual stimulus already be available from the related auditory stimulus? Alternatively, would an observer who saw both modalities together perhaps be able to make a better prediction of the MEG, on average, than would be possible if the modalities were not observed simultaneously?

This is conceptually the same question that is addressed with techniques such as RSA [26] or cross-decoding [27]. RSA determines similar representations by comparing the pairwise similarity structure in responses evoked by a stimulus set usually consisting of many exemplars with hierarchical categorical structure. If the pattern of pairwise relationships between stimulus-evoked responses is similar between two brain areas, it indicates there is a similarity in how the stimulus ensemble is represented. Cross-decoding works by training a classification or regression algorithm in one experimental condition or time region and then testing its performance in another experimental region or time region. If it performs above chance on the test set, this demonstrates some aspect of the representation in the data that the algorithm learned in the training phase is preserved in the second situation. Both these techniques address the same conceptual issue of representational similarity, which is measured with redundancy in the information theoretic framework, but have specific experimental design constraints and are usually used to compare different neural responses (recorded from different regions or time periods or with different experimental modalities). The information theoretic approach is more flexible and can be applied both to simple binary experimental conditions as well as continuous valued dynamic features extracted from complex naturalistic stimuli, such as those we consider here. Further, it allows us to study representational interactions between stimulus features (not only neural responses) and provides the ability to quantify synergistic as well as redundant interactions.

We can address this question with information theory through a quantity called “Interaction Information” [15, 58], which is defined as follows:

I(MEG;A;V)=I(MEG;[A,V])−I(MEG;A)−I(MEG;V) (1)

This quantifies the difference between the MI when the two modalities are observed together and the sum of the MI when each modality is considered alone.

Note that this is equivalent, with opposite sign, to a quantity called co-information. Considering co-information and thinking of information quantifying the size of sets that can be visualized in a Venn diagram, the terms I(MEG;A) + I(MEG;V) count the overall contribution of both variables but with any overlapping region counted twice. The term I(MEG;[A,V]) counts any overlapping region once and the nonoverlapping regions once each. So when subtracting the latter from the former, all that remains is the size of the overlapping region. This interpretation crucially depends on the property that MI is additive for independent variables, a property that is not shared by variance-based measures.

If the co-information overlap is positive, or equivalently interaction information (Eq 1) is negative, this indicates a redundant, or shared, representation. Some of what is learned about the neural response from the visual stimulus is already obtained from observation of the auditory stimulus. If the interaction information is positive, this indicates a synergistic representation. The two stimuli provide a better prediction when they are considered together than would be expected from observing each individually.

Interaction information is the difference between synergy and redundancy [19] and therefore measures a net effect. It is possible to have zero interaction information, even in the presence of strong redundant and synergistic interactions (for example, over different ranges of the stimulus space) that cancel out in the net value. The methodological problem of fully separating redundancy and synergy has recently been addressed with the development of a framework called the PID [19–22]. This provides a mathematical framework to obtain decomposition of MI into unique redundant and synergistic components. The PID requires a measure of information redundancy.

Here, we measure redundancy using a recently proposed method based on pointwise common change in surprisal; Iccs [15]. We use capital M,A,V to denote the MEG, auditory, and visual speech signals, respectively, and lower case letters to denote individual values of the same. Then, Iccs is defined as

$$\begin{array}{ll} I_{\text{ccs}}(M;A,V) & =\int_{m,a,v}\tilde{p}(m,a,v)i_{\text{ccs}}(m;a,v)\\ i_{\text{ccs}}(m;a,v) & =\{\begin{array}{c} c(m,a,v)\\ 0 \end{array}\begin{array}{c} \;\text{if}\;\text{sgn}(i(m;a))=\text{sgn}(i(m;v))=\text{sgn}(i(m;a,v))=\text{sgn}(c(m,a,v))\\ \text{otherwise} \end{array} \end{array}$$ (2)

where

i(m;a)=log2p(m,a)p(m)p(a)i(m;v)=log2p(m,v)p(m)p(v)i(m;a;v)=log2p˜(m,a,v)p(m)p˜(a,v)c(m,a,v)=i(m;a)+i(m;v)−i(m;a;v) (3)

Iccs exploits the additivity of local or pointwise information values (denoted with lower case *i*) to calculate, for each specific value of the variables considered, the overlap in pointwise information about the MEG signal that is shared between the auditory and visual speech signals. This is calculated using the local interaction information (the negative of which is called co-information and denoted c). The expectation of this pointwise overlap is then taken, in the same way MI is the expectation of pointwise values, but because of the sign conditions, only pointwise terms that unambiguously correspond to a redundant interaction are included. Full details of the measure are given in [15]. This is the only redundancy measure that corresponds to an intuitive notion of overlapping information content and is defined for more than two variables and for continuous systems. We use it here in a continuous Gaussian formulation together with the rank-normalization approach of GCMI. As there is no closed-form expression for Iccs in the case of Gaussian variables, we use Monte Carlo numerical integration. Note that the calculation requires a surrogate joint distribution over all three variables. Here, we use

$$\begin{array}{l} \tilde{P}(M,A,V)=\underset{Q\in \Delta_{P}}{\text{arg}\;\text{max}}H(Q)\\ \Delta_{P}=\left\{Q\in \Delta :Q(A,M)=P(A,M),Q(V,M)=P(V,M)\right\} \end{array}$$ (4)

This is the maximum entropy distribution that constrains the marginal distribution of each modality speech signal and MEG. The resulting redundancy measure is therefore invariant to the marginal dependence between auditory and visual signals (which differs here between conditions).

With a measure of redundant information in hand, the PID framework allows us to separate the redundant and synergistic contributions to the interaction information, as well as the unique information in each modality (Fig 1). For clarity, we restate the interpretation of these terms in this experimental context.

- Unique Information I_uni_(MEG;A): This quantifies that part of the MEG activity that can be explained or predicted only from the auditory speech envelope.
- Unique Information I_uni_(MEG;V): This quantifies that part of the MEG activity that can be explained or predicted only from the visual lip area.
- Redundancy I_red_(MEG;A,V): This quantifies the information about the MEG signal that is common to or shared between the two modalities. Alternatively, this quantifies the representation in the MEG of the variations that are common to both signals.
- Synergy I_syn_(MEG;A,V): This quantifies the extra information that arises when both modalities are considered together. It indicates that prediction of the MEG response is improved by considering the dynamic relationship between the two stimuli, over and above what could be obtained from considering them individually.

Conceptually, the redundancy is related to whether the information conveyed by A and V in individual samples is the same or different. If the variables are fully redundant, then this means either alone is enough to convey all the information about M (i.e., obtain an optimal prediction of M), and adding observation of the second modality has no benefit for prediction. The concept of synergy is related to whether A and V convey more information when observed simultaneously, so the prediction of M is enhanced by simultaneous observation of the values of A and V [15]. For example, if M is given by the difference between A and V at each sample, then observing either A or V alone tells little about the value of M, but observing them together completely determines it.

#### PID analysis

For brain signals, frequency-specific brain activation time series were computed by applying the beamformer coefficients to the MEG data filtered in the same frequency band (fourth-order Butterworth filter, forward and reverse, center frequency ±2 Hz). The auditory and visual speech signals were filtered in the same frequency band (5 ± 2 Hz; 3–7 Hz) after we checked the dependencies between matching or nonmatching auditory and visual speeches used in the present study (Fig 2A). MEG signals were shifted by 100 ms as in previous studies [5, 18] to compensate for delays between stimulus presentation and cortical responses. Then, each map of PID was computed using these auditory and visual speech signals and source-localized brain signal for each voxel and each frequency band.

As described above (MI between auditory and visual speech signals), the complex spectra obtained from the Hilbert transform were amplitude normalized, and the real and imaginary parts were each rank-normalized. The covariance matrix of the full 6-dimensional signal space was then computed, which completely describes the Gaussian-Copula dependence between the variables. The PID was applied with redundancy measured by pointwise common change in surprisal (Iccs) [15] for Gaussian variables as described above.

This calculation was performed independently for each voxel, resulting in volumetric maps for the four PID terms (redundant information, unique information of auditory speech, unique information of visual speech, synergistic information) for each frequency band in each individual. This computation was performed for all experimental conditions: “All congruent,” “All incongruent,” and “AV congruent.”

In addition, surrogate maps were created by computing the same decomposed information maps between brain signals and time-shifted speech signals for each of the four experimental conditions in each individual. Visual speech signals were shifted for 30 s, and auditory speech signals were shifted for 60 s. These surrogate data provide an estimate of each information map that can be expected by chance for each condition. These surrogate data are not used to create a null distribution but to estimate analysis bias at the group level. The surrogate data are used in analysis for Figs 1B–1D, 5C and 5D, and S1, S2 and S4 Figs.

#### Statistics

Group statistics was performed on the data of all 44 participants in FieldTrip. First, individual volumetric maps for each calculation (MI, Unique Information, Redundancy, Synergy) were smoothed with a 10 mm Gaussian kernel. Then, they were subjected to dependent *t* statistics using nonparametric randomization (Monte Carlo randomization) for comparisons between experimental conditions or to surrogate data. Results are reported after multiple comparison correction was performed using FDR [59].

For the maps relating synergistic and redundant PID relevant for behavior, each information map (unique, redundant, and synergistic map) was subjected to regression analysis. In the regression analysis, we detected brain regions that were positively correlated to comprehension accuracy using nonparametric randomization (Monte Carlo randomization). Then, regression *t* maps were converted to standard *Z*-map (*Z*-transformation) and subtracted between conditions (*P* < 0.005).

## Supporting information

S1 Fig Neural decomposition of natural audiovisual speech (‘All congruent’ condition). In order to understand multisensory representational interactions in the brain during processing of natural audiovisual speech, we first define characteristics of decomposed information in ‘All congruent’ condition. To observe overall patterns in each information map, we first normalized each information map by time-shifted surrogate map at individual level, then averaged across subjects. **(A)** This multisensory audiovisual MI (total mutual information I(MEG;A,V)) includes unique unimodal as well as redundant and synergistic multisensory effects, which we can separate with the PID. The total mutual information map shows multimodal stimulus entrainment in bilateral auditory/temporal areas and to lesser extent in visual cortex. **(B)** Auditory unique information I(MEG;A) is present in bilateral auditory areas, where it accounts for a large proportion of the total mutual information I(MEG;A,V). **(C)** Visual unique information I(MEG;V) is present in both visual and auditory areas, but overall visual entrainment is weaker than auditory entrainment. **(D)** We suspect that the auditory-temporal involvement in the visual unique information might due to the correlation between auditory and visual speech signals (Fig 2A), so we computed visual unique information in ‘All incongruent’ condition where auditory and visual speech do not match. As expected, it is only present in visual areas. Please note that these figures represent grand averages and not statistical maps. To further investigate each information of PID, we used predefined ROI maps from SPM Anatomy Toolbox (version 2.1) [1 in S1 References] and Automated Anatomical Labeling (AAL) [2 in S1 References]. SPM Anatomy Toolbox provides probabilistic cytoarchitectonic maps which provides stereotaxic information on the location and variability of cortical areas in the MNI (Montreal Neurological Institute) space. AAL maps provide anatomical parcellation of the spatially normalized single-subject high-resolution T1 of MNI space. Both ROI toolbox provide complementary ROI maps to each other, so that we used both toolboxes. We were interested in each decomposed PID profile in natural speech condition in four main areas: Auditory/Temporal, Visual, Motor/Sensory, Language-related areas. Auditory/Temporal area (purple) includes primary auditory cortex (TE 1.0, TE 1.1, TE 1.2), higher auditory cortex (TE 3), superior temporal gyrus (STG), superior temporal pole (STG p), middle temporal gyrus (MTG), middle temporal pole (MTG p), inferior temporal gyrus (ITG). Visual area (blue) includes BA17/V1 (hOC1), BA18/V2 (hOC2), dorsal extrastriate cortex (hOC3d/hOC4d), ventral extrastriate cortex (hOC3v/hOC4v), lateral occipital cortex (hOc4la, hOc4lp), V5/MT+ area (hOc5). Motor/Sensory area (brown) includes Areas 4a and 4p, supplementary motor area (SMA), and primary somatosensory cortex Areas 1, 2, 3a, 3b. Language-related area (red) includes BA44, BA45, Inferior frontal opercular part (Inf Oper), Inferior frontal triangular part (Tri Oper), Rolandic operculum (Rol Oper), supramarginal gyrus (SMG), and angular gyrus (AG). We first transformed the dimension of each ROI map to the dimension of our source space data, then we extracted each information (unique unimodal information for auditory and visual speech, redundancy and synergy) of bandpass-filtered (low frequencies 1–7 Hz) phase data from each ROI and then each information value was averaged within the ROI. This was performed for ‘All congruent’ condition and time-shifted surrogate data. Each information data was averaged across all subjects after subtracted by surrogate data within individual (mean ± s.e.m). Data shown per each hemisphere (LH, RH). Statistics compared to the time-shifted surrogate data was also performed and shown with asterisk in each bar when it is significant (paired two-sided *t*-test; df: 43; *P* < 0.05). First for unimodal unique information (UI-A, UI-V), as expected, auditory unique information **(E, F)** showed strong unique information in primary auditory cortices while visual unique information **(G, H)** showed strong unique information in visual cortices as well as auditory/temporal areas (uncorrected statistics). This auditory/temporal representation of visual unique information is interesting (see also C) and might be due to the correlated features of audiovisual speech signals when they are congruent (Fig 2A) as in the ‘All congruent’ condition. This is plausible because when we analysed visual unique information for ‘All incongruent’ condition where auditory and visual speeches are incongruent, it only showed visual areas (D). There have been studies on the auditory representation of visual speech demonstrating auditory cortical activation during silent lipreading [3 in S1 References], and other studies showing primary auditory [4, 5 in S1 References] or auditory/temporal association cortices [6–8 in S1 References] when audiovisual stimuli are congruent. The underlying mechanisms should be further elucidated, but this is likely due to feedback processes in the brain related to multisensory representation of congruent (thus correlated) audiovisual speech [9, 10 in S1 References]. Next, for Redundancy **(I, J)** and Synergy **(K, L)**, overall redundant information in both hemispheres is strong. This was expected considering the same audiovisual inputs in this condition (‘All congruent’). In auditory/temporal areas, both redundant and synergistic information were significantly different from time-shifted surrogate data. This pattern is the same in both hemispheres. However, in visual areas, only redundant information is significant when compared to time-shifted surrogate data whereas nearly none of synergistic information in visual cortices remain significant. In motor/sensory and language-related areas, redundant information is strongly significant in both hemispheres. Synergistic information in inferior frontal regions was also significant with more left-lateralized pattern. It should be noted that this pattern arises when perceived speech is natural unlike when task is challenging as in ‘AV congruent’ condition (the main analysis). The underlying data for this figure are available from the Open Science Framework (https://osf.io/hpcj8/).
(7Z) Click here for additional data file.

S2 Fig Left pSTG and left motor cortex differentially predict visual speech. A potential benefit of speech-entrained brain activity is the facilitation of temporal prediction of upcoming speech. We therefore investigated to what extent the different integration mechanisms in pSTG and motor cortex (reflected by differences in redundancy versus synergy) lead to differences in prediction. Since informativeness changed most strongly for the visual (speech) input signal (informative for ‘AV congruent’, less informative for ‘All congruent’), we expected strongest prediction effects for visual speech.
**Delayed Mutual Information analysis.** We used Delayed Mutual Information to investigate to what extent brain areas predict upcoming auditory or visual speech. Delayed mutual information refers to mutual information between two signals offset with different delays. If there is significant MI between brain activity at one time, and the speech signal at a later time, this shows that brain activity contains information about the future of the speech signal. Directed Information or Transfer Entropy [11, 12 in S1 References], is based on the same principle but additionally conditions out the past of the speech signal, to ensure the delayed interaction is providing new information over and above that available in the past of the stimulus. Here, since the delayed MI peaks are clear and well isolated from the 0 lag we present the simpler measure, but transfer entropy calculations revealed similar effects (results not shown). By means of delayed MI, we investigated prediction mechanism between theta phase in each brain area and later theta phase in visual speech. We tested delays from 0 ms to 500 ms in steps of 20 ms and then averaged the values across these delays. Interestingly, prediction of visual speech varied in both brain areas between conditions, but in different ways. Left pSTG predicts visual speech stronger in ‘All congruent’ and ‘AV congruent’ conditions than in incongruent condition (**A**; *t* = 2.99, *P* = 0.004 in ‘All congruent’ > ‘All incongruent’; *t* = 2.32, *P* = 0.02 in ‘AV congruent’ > ‘All incongruent’). Left motor cortex predicts visual speech stronger for ‘AV congruent’ than ‘All congruent’ (**B**; attention effect; *t* = 2.24, *P* = 0.03). When we unfolded these patterns in the temporal domain more interesting pattern emerged. The prediction mechanism in left pSTG operates in shorter temporal delays of 150–300 ms (**C**; *P* < 0.05), but left motor cortex is involved in longer temporal delays of 350 ms and above (**D**; *P* < 0.05). These findings suggest that left pSTG is mostly sensitive to congruent audiovisual speech (as demonstrated by redundancy in Fig 4) and best predicts visual speech when congruent audiovisual speech is available in the absence of distracting input. This happens fast at shorter delays with visual speech. However, this pattern is different for left motor cortex. Here, we see better prediction in the ‘AV congruent’ condition, when visual speech information is informative, attended and useful to resolve a challenging listening task. Thus it has rather slow temporal dynamics at delays greater than 350 ms. The prediction of auditory speech was not different between conditions. This is expected because the level of auditory attention is similar across conditions. Overall, this suggests that integration mechanisms in left pSTG are optimized for congruent audiovisual speech. This is consistent with results in Figs 4 and 5 that show prominent redundancy in left pSTG. Left motor cortex instead seems to play an important role when greater attentional efforts are required and potential conflicts need to be resolved (as is the case for ‘AV congruent’ condition). The underlying data for this figure are available from the Open Science Framework (https://osf.io/hpcj8/).
(TIF)

S3 FigLocal maps of redundancy and synergy in searching for mechanisms of AV speech interaction.This figure presents more detailed results from an analysis of interactions between auditory and visual speech signals as predictors of MEG signal in left superior temporal gyrus (pSTG) and left motor cortex. The selection of regions of interest was based on the results presented in Figs 3–5 that left pSTG features largely redundant interactions whereas left motor areas show predominantly synergistic interactions. As described in the Methods section, we estimate information quantities using Gaussian-Copula Mutual Information (GCMI) [42] which provides a robust semi-parametric lower bound estimator of mutual information, by combining the statistical theory of copulas with the closed form solution for the entropy of Gaussian variables. Crucially, this method performs well for higher dimensional responses as required for measuring three-way statistical interactions and allows estimation over circular variables like phase. Complex spectra from Hilbert-transformed signals of auditory speech, visual speech and each brain region were amplitude normalized, and the real and imaginary parts were rank-normalized. The covariance matrix describing Gaussian-Copula dependence was computed. Information and PID values are expectations over the space of joint values. To get more insight into the mechanisms underlying the information theoretic quantification, we can directly visualise the values which are summed in the expectation, often called local values [14 in S1 References]. While the main analysis involved 2D Hilbert transformed signals, for ease of visualisation we here consider just the 1D bandpass filtered signal. Joint MI can be written as: $I\left(A,V;MEG\right)=\;{∭}_{A,V,MEG}p\left(a,v,meg\right)i\left(a,v;meg\right)da\;dv\;dmeg$ where i(a,v;meg)=log2p(a,v,meg)p(a,v)p(meg) is the local information. We plot here the combined term *pi*, the local information quantity multipled by the probability of those values. This breaks down the overall quantity into the individual values which are integrated over the space. After copula normalization of a signal 0 corresponds to the median amplitude, so positive values on the x- and y-axis correspond to amplitude values above the median for auditory and visual speech signals, respectively. Here we study Mutual Information (MI) between auditory and visual speech for both regions of interest (upper row; **A, B, E, F**) and redundancy for left pSTG and synergy for left motor cortex (bottom row) averaged across all participants. In the ‘L pSTG’ plot, when MEG response < 0 **(C)**, the redundancy comes from above median values of both auditory and visual speech. This shows that when both auditory and visual speech signals are high (above median), they redundantly suggest that MEG response is below the median value in the band. However, when both auditory and visual speech signals are below their median values, they redundantly suggest an above median MEG response **(D)**. The ‘L Motor Cortex’ plot shows that when auditory and visual signals have opposite signs (i.e. above median value in one signal occurring with a below median value of the other signal, red diagonal nodes) **(G,H)** they synergistically inform about a co-occurring MEG response value. That is, knowing that A is high and V is low together (or know that V is high and A is low together), provides a better prediction of a specific MEG response value than would be expected if the evidence was combined independently. Here the negative node (blue) indicates a negative synergistic contribution to mutual information (sometimes called misinformation). Above/below median values can be interpreted as loud/quiet auditory speech and large/small lip movement, so low sound amplitude combined with small lip movement can produce larger response in the left pSTG whereas high sound amplitude combined with large lip movement can produce smaller response in the left pSTG. This seems highly plausible mechanism given the left pSTG’s role in AV integration that it has greater involvement when both speech are not physically strong enough. However, synergy shows a different pattern that regardless of high or low MEG response in the left motor cortex, the combination of low sound amplitude and large lip movement, or combination of high sound amplitude and small lip movement can produce synergistic information. This suggests synergistic information arises when the interaction comes out of unbalanced features of predictors that are complementary to each other. The underlying data for this figure are available from the Open Science Framework (https://osf.io/hpcj8/).(TIF)

S4 Fig Synergy between brain regions predictive of visual speech. To better understand the integration mechanism of audiovisual speech processing observed in redundant and synergistic interaction between multisensory speech signals predictive of brain activity, we computed PID differently in which redundant and synergistic interaction between brain regions predictive of speech signals (auditory or visual).
**Selection of brain regions.** We selected eight brain regions to test a predictive mechanism, i.e., does MEG activity predict upcoming speech. Regions were selected from the maximum coordinates of contrast for attention and congruence effects shown in Figs 4 and 5. The abbreviation used in the nodes in the Figures, MNI coordinates, Talairach coordinates [15 in S1 References] and Brodmann area (BA) are shown in the parenthesis: Left auditory cortex (A1; MNI = [–36–24 8]; TAL = [-35.6–22.9 8.5]; BA 41/22), left visual cortex (V1; MNI = [–28–88–8]; TAL = [-27.7–85.6–2.5]; BA 18), left posterior superior temporal gyrus (pSTG; MNI = [–60–24 0]; TAL = [-59.4–23.3 1.2]; BA 21/22), left motor cortex (M1; MNI = [–44 0 64]; TAL = [-43.6 2.9 58.8]; BA 6), left supplementary motor area (SMA; MNI = [–4 0 48]; TAL = [-4.0 2.2 44.1]; BA 6), left inferior frontal gyrus (IFG; MNI = [–64 16 24]; TAL = [-63.4 16.6 21.3]; BA 44/45), right inferior frontal gyrus (IFG; MNI = [60 8 16]; TAL = [59.4 8.5 14.3]; BA 44), left precuneus (Prec; MNI = [–4–72 64]; TAL = [-4–66.8 62.3]; BA 7).
**Partial Information Decomposition (PID) analysis predictive of speech.** The PID analysis described above was computed to investigate cross-modal AV representational interactions in an individual brain region. But both PID and interaction information can be applied also to consider representational interactions between two brain regions to a single stimulus feature (as RSA is normally applied). To understand representational interactions between brain regions predictive of speech signals, we computed PID values with activity from two brain regions as the predictor variables and a unimodal speech signal (auditory or visual) as a target variable. For this, we used the eight brain regions (see above) and computed the PID for each pair of eight regions predictive of visual speech or auditory speech. This resulted in 28 pairwise computations (n(n-1)/2). Here, redundant information between two brain regions means they both provide the same prediction of the upcoming speech signal. A synergistic interaction demonstrates that the particular dynamic relationship between the neural activity in the two regions is itself predictive of speech, in a way that the direct recorded MEG in each region alone is not. The signals at the maximum coordinates were extracted from each region, PID was computed for each pair of brain regions predictive of auditory or visual speech signals. Each condition was compared to time-shifted surrogate data and between conditions. We found interesting results for synergistic interaction (but not for redundant information) between brain regions on visual speech for attention effect (‘AV congruent’ > ‘All congruent’). **(A)** ‘AV congruent’ vs. surrogate data. Synergistic interaction between IFG (L)–M1, IFG (L)–SMA, A1–V1, A1–IFG (R), pSTG–IFG (R), pSTG–V1 were significant when predictive of visual speech. **(B)** ‘All congruent’ vs. surrogate data. Synergistic interaction between A1–V1, pSTG–V1 were shown to be predictive of visual speech. **(C)** ‘AV congruent’ vs. ‘All congruent’ (attention). When these two conditions were compared directly, synergistic interaction between IFG (L)–M1, IFG (L)–SMA, IFG (L)–A1, IFG (L)–Precuneus, SMA–Precuneus were observed to be predictive of visual speech (paired two-sided *t*-test; *P* < 0.05). However, we could not find any significant interaction (either redundancy or synergy) between these regions predictive of auditory speech. These results suggest that synergistic information interaction between the regions centering around left inferior frontal gyrus (BA44/BA45) and motor areas, which matches dorsal stream in speech processing [24], plays important role in attention to speech particularly visual speech when the task is challenging. The underlying data for this figure are available from the Open Science Framework (https://osf.io/hpcj8/).
(TIF)

S5 Fig Attention to visual speech revealed by analysis of eye fixation. Participants were carefully instructed to fixate on the speaker’s mouth in all experimental conditions and we monitored participants’ eye movement using an eye tracker (see Materials and methods) to ensure that they fixate on the speaker’s lip movements. In order to investigate attention to visual speech, we analysed the simultaneously recorded eye tracking data. **(A)** We first constructed 2D-histograms of fixation position throughout the recording sessions (while participants viewed the speaker’s face) for each participant and each experimental condition. These histograms support the fact that participants followed instructions and fixated on the speaker’s mouth. Compliance with the instructions means that the visual information was available to them and it seems unlikely that this information was not used in the case of an interfering auditory stimulus (‘AV congruent’ condition). **(B)** We further analysed the 2D distribution of fixations by fitting Gaussian functions along the horizontal and vertical dimension for each participant and each experimental condition. Statistical comparison (*t*-test) of the width of the Gaussian function for horizontal dimension revealed a significant difference between conditions with congruent auditory and visual stimuli compared to incongruent auditory and visual stimuli (paired two sided *t*-test, df: 43; ‘AV congruent’ vs. ‘All incongruent’: *t* = -2.94, *P* = 0.005; ‘All congruent’ vs. ‘All incongruent’: *t* = -2.39, *P* = 0.02). Congruent AV stimuli had a significantly lower width (more narrow distribution) indicating a more focussed fixation on the mouth compared to incongruent AV stimuli (where visual stimulus was not informative). This result is not an unambiguous proof but it suggests that the informative visual information is attended and used by the participant. The underlying data for this figure are available from the Open Science Framework (https://osf.io/hpcj8/).
(TIF)

S1 References Reference list for Supporting Information. (DOCX)

## Abbreviations

BOLD: blood oxygen level–dependent
ECG: electrocardiographic
EOG: electrooculographic
FDR: false discovery rate
fMRI: functional magnetic resonance imaging
FOV: field of view
fps: frame per second
GCMI: Gaussian-Copula Mutual Information
ICA: independent component analysis
MEG: magnetoencephalography
MI: mutual information
MNI: Montreal Neurological Institute
PID: partial information decomposition
pSTG/S: posterior superior temporal gyrus/sulcus
pSTS: posterior superior temporal sulcus
RSA: representational similarity analysis
STG/S: superior temporal gyrus/sulcus
TMS: transcranial magnetic stimulation
