# Supplementary figures and images for: Representational interactions during audiovisual speech entrainment: Redundancy in left posterior superior temporal gyrus and synergy in left motor cortex
Source: PLoS Biol. 2018 Aug 6;16(8):e2006558. doi: 10.1371/journal.pbio.2006558 (PMC6095613; doi:10.1371/journal.pbio.2006558)

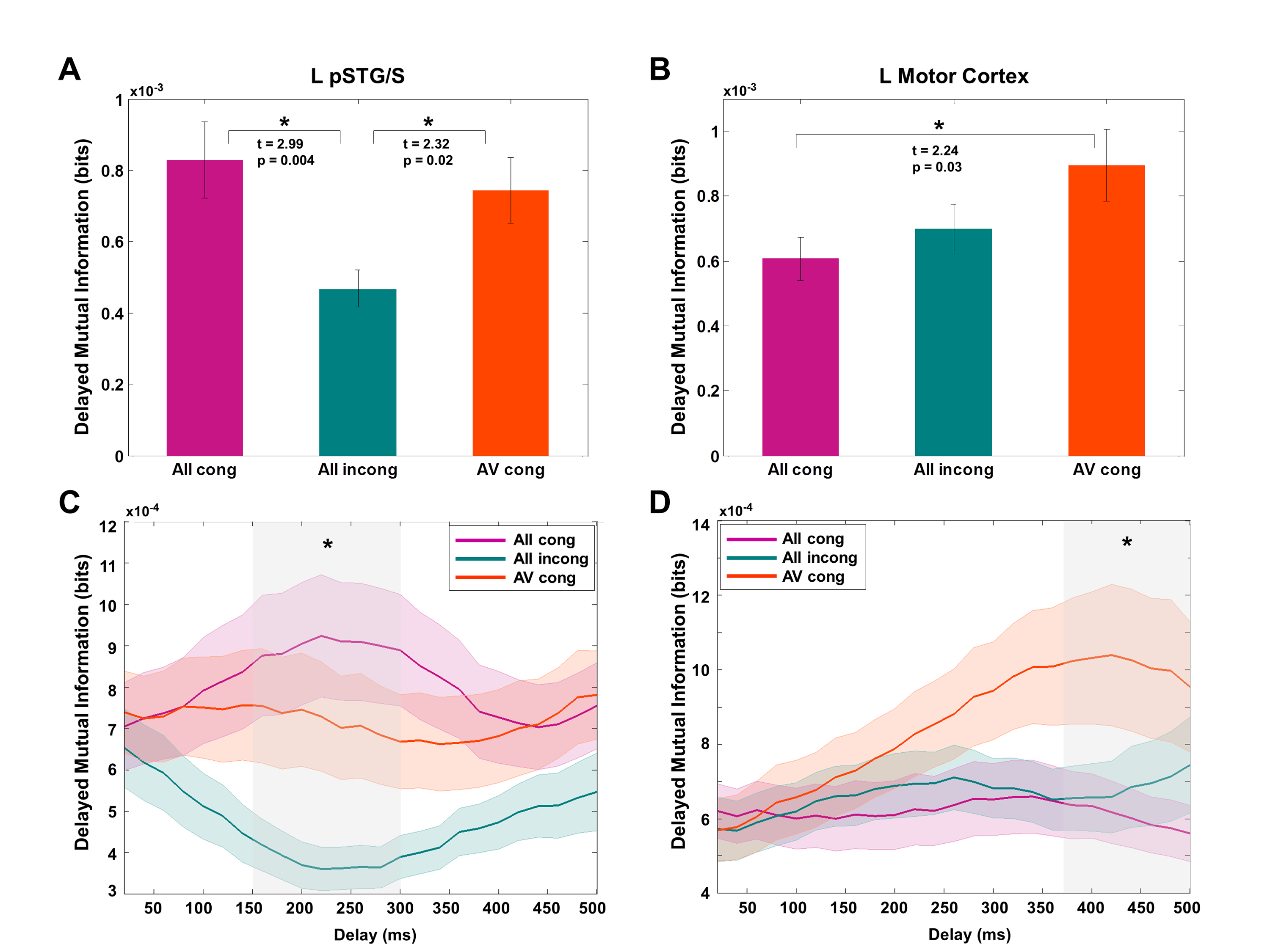

Supplement: S2 Fig — A potential benefit of speech-entrained brain activity is the facilitation of temporal prediction of upcoming speech. We therefore investigated to what extent the different integration mechanisms in pSTG and motor cortex (reflected by differences in redundancy versus synergy) lead to differences in prediction. Since informativeness changed most strongly for the visual (speech) input signal (informative for ‘AV congruent’, less informative for ‘All congruent’), we expected strongest prediction effects for visual speech. Delayed Mutual Information analysis. We used Delayed Mutual Information to investigate to what extent brain areas predict upcoming auditory or visual speech. Delayed mutual information refers to mutual information between two signals offset with different delays. If there is significant MI between brain activity at one time, and the speech signal at a later time, this shows that brain activity contains information about the future of the speech signal. Directed Information or Transfer Entropy [11, 12 in S1 References], is based on the same principle but additionally conditions out the past of the speech signal, to ensure the delayed interaction is providing new information over and above that available in the past of the stimulus. Here, since the delayed MI peaks are clear and well isolated from the 0 lag we present the simpler measure, but transfer entropy calculations revealed similar effects (results not shown). By means of delayed MI, we investigated prediction mechanism between theta phase in each brain area and later theta phase in visual speech. We tested delays from 0 ms to 500 ms in steps of 20 ms and then averaged the values across these delays. Interestingly, prediction of visual speech varied in both brain areas between conditions, but in different ways. Left pSTG predicts visual speech stronger in ‘All congruent’ and ‘AV congruent’ conditions than in incongruent condition (A; t = 2.99, P = 0.004 in ‘All congruent’ > ‘All incongruent’; t = [file pbio.2006558.s002.tif]

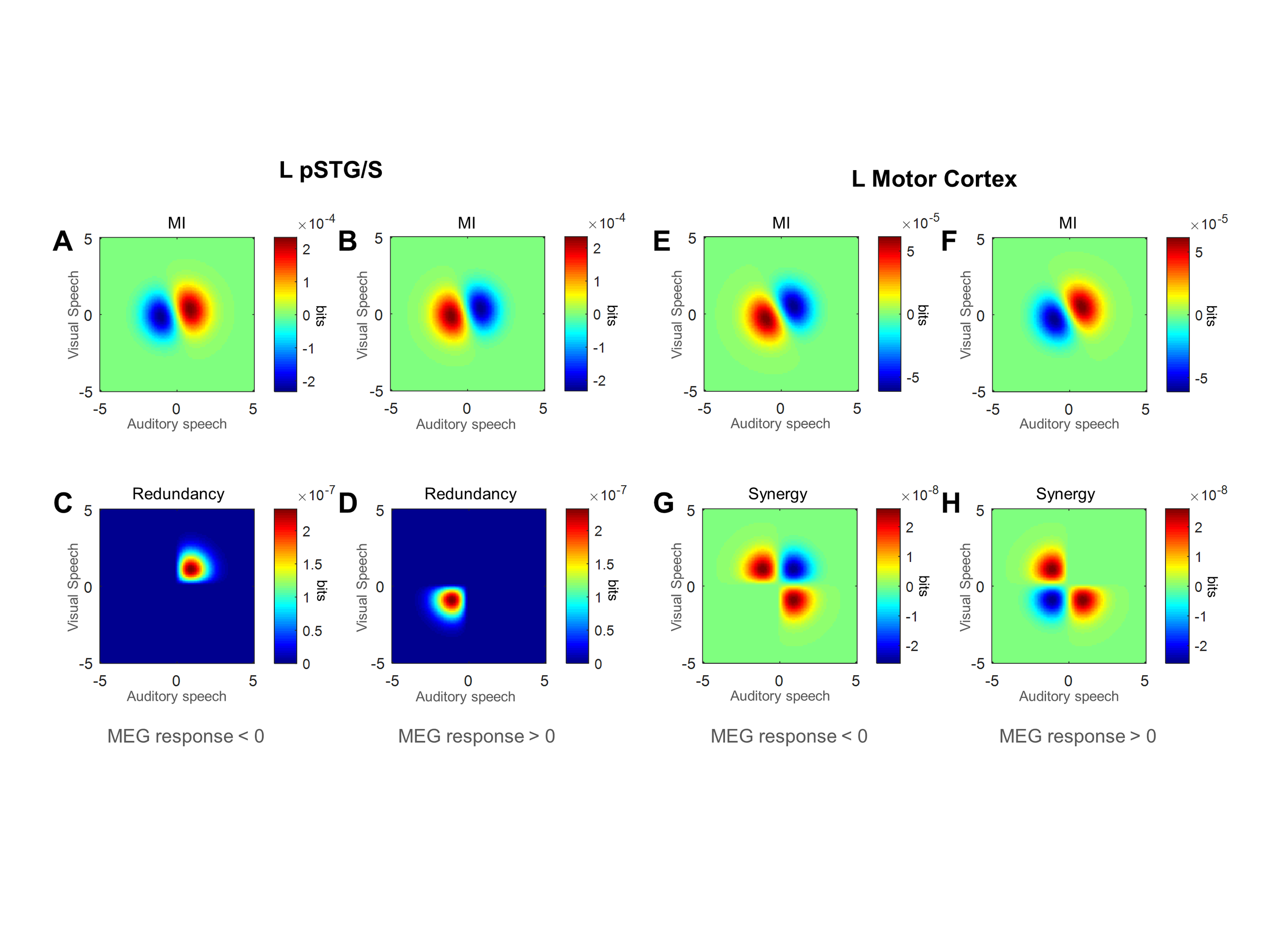

Supplement: S3 Fig — This figure presents more detailed results from an analysis of interactions between auditory and visual speech signals as predictors of MEG signal in left superior temporal gyrus (pSTG) and left motor cortex. The selection of regions of interest was based on the results presented in Figs 3–5 that left pSTG features largely redundant interactions whereas left motor areas show predominantly synergistic interactions. As described in the Methods section, we estimate information quantities using Gaussian-Copula Mutual Information (GCMI) [42] which provides a robust semi-parametric lower bound estimator of mutual information, by combining the statistical theory of copulas with the closed form solution for the entropy of Gaussian variables. Crucially, this method performs well for higher dimensional responses as required for measuring three-way statistical interactions and allows estimation over circular variables like phase. Complex spectra from Hilbert-transformed signals of auditory speech, visual speech and each brain region were amplitude normalized, and the real and imaginary parts were rank-normalized. The covariance matrix describing Gaussian-Copula dependence was computed. Information and PID values are expectations over the space of joint values. To get more insight into the mechanisms underlying the information theoretic quantification, we can directly visualise the values which are summed in the expectation, often called local values [14 in S1 References]. While the main analysis involved 2D Hilbert transformed signals, for ease of visualisation we here consider just the 1D bandpass filtered signal. Joint MI can be written as: I(A,V;MEG)=∭A,V,MEGp(a,v,meg)i(a,v;meg)dadvdmeg where i(a,v;meg)=log2p(a,v,meg)p(a,v)p(meg) is the local information. We plot here the combined term pi, the local information quantity multipled by the probability of those values. This breaks down the overall quantity into the individual values which are integrated over the space. After co [file pbio.2006558.s003.tif]

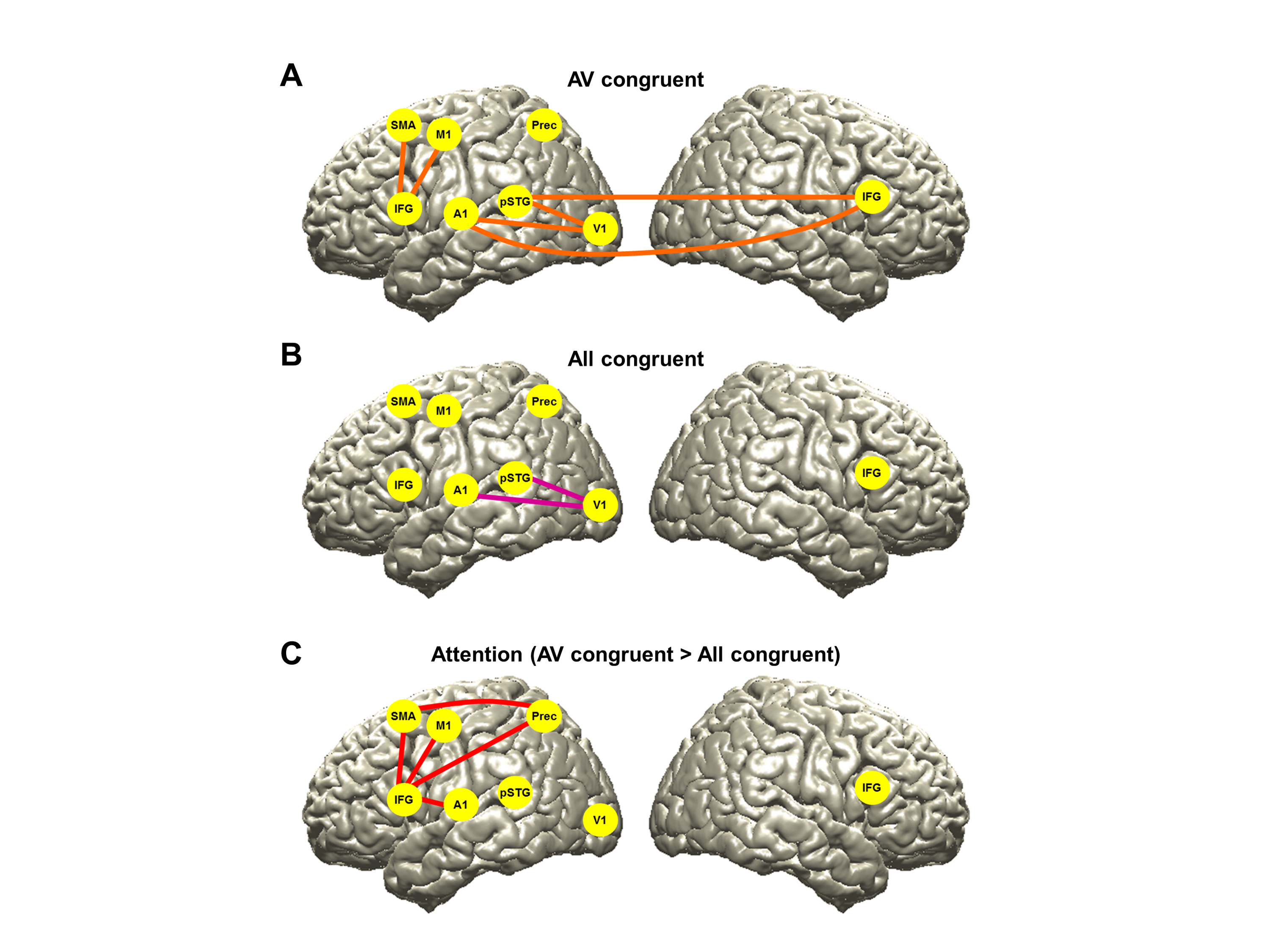

Supplement: S4 Fig — To better understand the integration mechanism of audiovisual speech processing observed in redundant and synergistic interaction between multisensory speech signals predictive of brain activity, we computed PID differently in which redundant and synergistic interaction between brain regions predictive of speech signals (auditory or visual). Selection of brain regions. We selected eight brain regions to test a predictive mechanism, i.e., does MEG activity predict upcoming speech. Regions were selected from the maximum coordinates of contrast for attention and congruence effects shown in Figs 4 and 5. The abbreviation used in the nodes in the Figures, MNI coordinates, Talairach coordinates [15 in S1 References] and Brodmann area (BA) are shown in the parenthesis: Left auditory cortex (A1; MNI = [–36–24 8]; TAL = [-35.6–22.9 8.5]; BA 41/22), left visual cortex (V1; MNI = [–28–88–8]; TAL = [-27.7–85.6–2.5]; BA 18), left posterior superior temporal gyrus (pSTG; MNI = [–60–24 0]; TAL = [-59.4–23.3 1.2]; BA 21/22), left motor cortex (M1; MNI = [–44 0 64]; TAL = [-43.6 2.9 58.8]; BA 6), left supplementary motor area (SMA; MNI = [–4 0 48]; TAL = [-4.0 2.2 44.1]; BA 6), left inferior frontal gyrus (IFG; MNI = [–64 16 24]; TAL = [-63.4 16.6 21.3]; BA 44/45), right inferior frontal gyrus (IFG; MNI = [60 8 16]; TAL = [59.4 8.5 14.3]; BA 44), left precuneus (Prec; MNI = [–4–72 64]; TAL = [-4–66.8 62.3]; BA 7). Partial Information Decomposition (PID) analysis predictive of speech. The PID analysis described above was computed to investigate cross-modal AV representational interactions in an individual brain region. But both PID and interaction information can be applied also to consider representational interactions between two brain regions to a single stimulus feature (as RSA is normally applied). To understand representational interactions between brain regions predictive of speech signals, we computed PID values with activity from two brain regions as the predictor variables [file pbio.2006558.s004.tif]

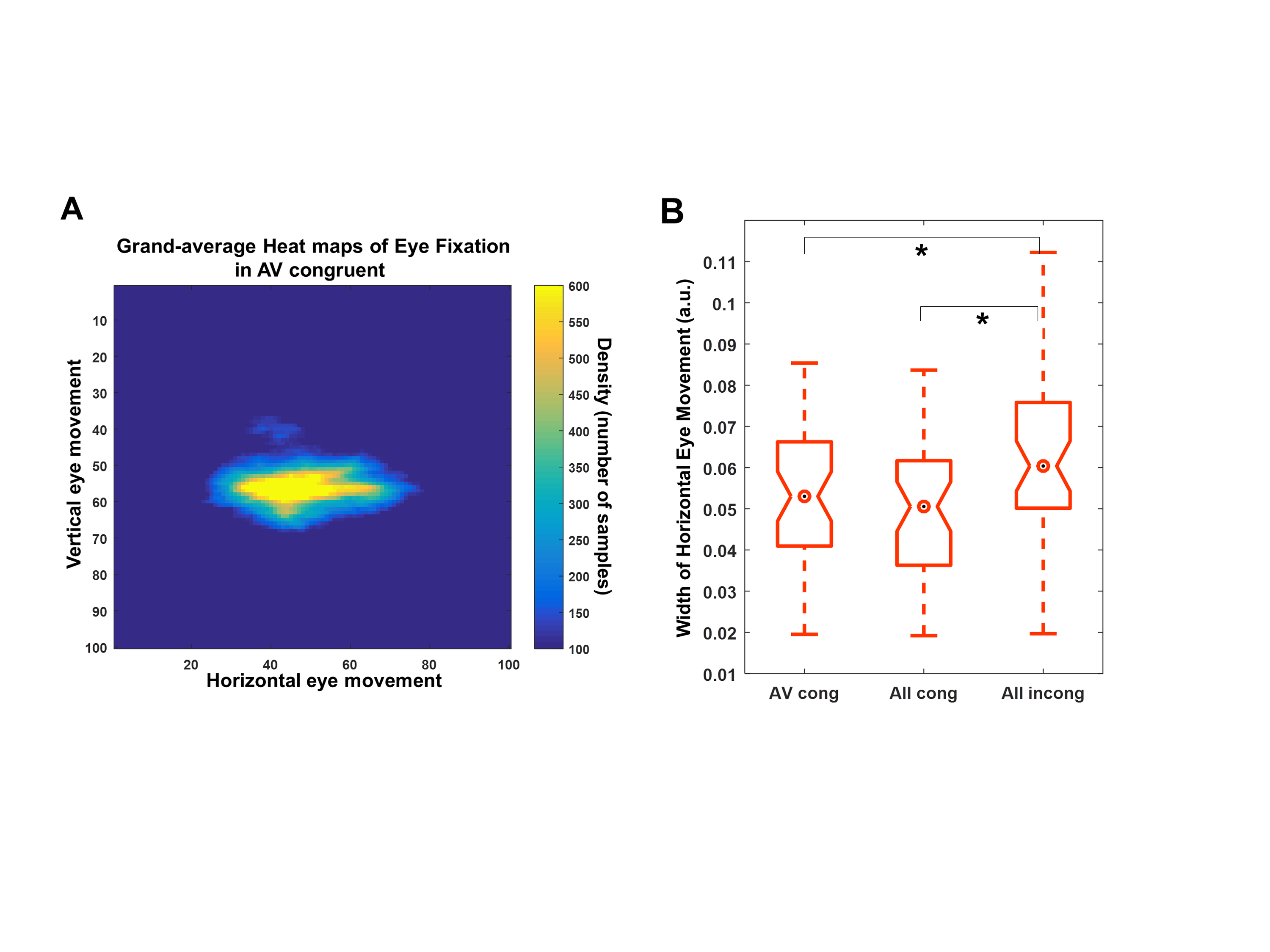

Supplement: S5 Fig — Participants were carefully instructed to fixate on the speaker’s mouth in all experimental conditions and we monitored participants’ eye movement using an eye tracker (see Materials and methods) to ensure that they fixate on the speaker’s lip movements. In order to investigate attention to visual speech, we analysed the simultaneously recorded eye tracking data. (A) We first constructed 2D-histograms of fixation position throughout the recording sessions (while participants viewed the speaker’s face) for each participant and each experimental condition. These histograms support the fact that participants followed instructions and fixated on the speaker’s mouth. Compliance with the instructions means that the visual information was available to them and it seems unlikely that this information was not used in the case of an interfering auditory stimulus (‘AV congruent’ condition). (B) We further analysed the 2D distribution of fixations by fitting Gaussian functions along the horizontal and vertical dimension for each participant and each experimental condition. Statistical comparison (t-test) of the width of the Gaussian function for horizontal dimension revealed a significant difference between conditions with congruent auditory and visual stimuli compared to incongruent auditory and visual stimuli (paired two sided t-test, df: 43; ‘AV congruent’ vs. ‘All incongruent’: t = -2.94, P = 0.005; ‘All congruent’ vs. ‘All incongruent’: t = -2.39, P = 0.02). Congruent AV stimuli had a significantly lower width (more narrow distribution) indicating a more focussed fixation on the mouth compared to incongruent AV stimuli (where visual stimulus was not informative). This result is not an unambiguous proof but it suggests that the informative visual information is attended and used by the participant. The underlying data for this figure are available from the Open Science Framework (https://osf.io/hpcj8/). (TIF) [file pbio.2006558.s005.tif]
