# Supplementary material for: Representational interactions during audiovisual speech entrainment: Redundancy in left posterior superior temporal gyrus and synergy in left motor cortex
Source: PLoS Biol. 2018 Aug 6;16(8):e2006558. doi: 10.1371/journal.pbio.2006558 (PMC6095613; doi:10.1371/journal.pbio.2006558)
Supplement: S1 References — (DOCX) [file pbio.2006558.s006.docx]

**Supplementary Information References**

1. Eickhoff SB, Stephan KE, Mohlberg H, Grefkes C, Fink GR, Amunts K, et al. A new SPM toolbox for combining probabilistic cytoarchitectonic maps and functional imaging data. NeuroImage. 2005;25(4):1325-35. doi: 10.1016/j.neuroimage.2004.12.034. PubMed PMID: 15850749.

2. Tzourio-Mazoyer N, Landeau B, Papathanassiou D, Crivello F, Etard O, Delcroix N, et al. Automated anatomical labeling of activations in SPM using a macroscopic anatomical parcellation of the MNI MRI single-subject brain. NeuroImage. 2002;15(1):273-89. doi: 10.1006/nimg.2001.0978. PubMed PMID: 11771995.

3. Crosse MJ, ElShafei HA, Foxe JJ, Lalor EC, editors. Investigating the Temporal Dynamics of Auditory Cortical Activation to Silent Lipreading. 7th Annual International IEEE EMBS Conference on Neural Engineering; 2015; Montpellier, France.

4. Okada K, Venezia JH, Matchin W, Saberi K, Hickok G. An fMRI Study of Audiovisual Speech Perception Reveals Multisensory Interactions in Auditory Cortex. PLoS One. 2013;8(6):e68959. doi: 10.1371/journal.pone.0068959. PubMed PMID: 23805332; PubMed Central PMCID: PMCPMC3689691.

5. Pekkola J, Ojanen V, Autti T, Jaaskelainen IP, Mottonen R, Tarkiainen A, et al. Primary auditory cortex activation by visual speech: an fMRI study at 3 T. Neuroreport. 2005;16(2):125-8. PubMed PMID: 15671860.

6. Bernstein LE, Auer ET, Jr., Moore JK, Ponton CW, Don M, Singh M. Visual speech perception without primary auditory cortex activation. Neuroreport. 2002;13(3):311-5. PubMed PMID: 11930129.

7. Besle J, Fischer C, Bidet-Caulet A, Lecaignard F, Bertrand O, Giard MH. Visual activation and audiovisual interactions in the auditory cortex during speech perception: intracranial recordings in humans. J Neurosci. 2008;28(52):14301-10. doi: 10.1523/JNEUROSCI.2875-08.2008. PubMed PMID: 19109511.

8. Calvert GA, Campbell R. Reading speech from still and moving faces: the neural substrates of visible speech. J Cogn Neurosci. 2003;15(1):57-70. doi: 10.1162/089892903321107828. PubMed PMID: 12590843.

9. Bernstein LE, Liebenthal E. Neural pathways for visual speech perception. Front Neurosci. 2014;8:386. doi: 10.3389/fnins.2014.00386. PubMed PMID: 25520611; PubMed Central PMCID: PMCPMC4248808.

10. Schroeder CE, Lakatos P, Kajikawa Y, Partan S, Puce A. Neuronal oscillations and visual amplification of speech. Trends in cognitive sciences. 2008;12(3):106-13. doi: 10.1016/j.tics.2008.01.002. PubMed PMID: 18280772; PubMed Central PMCID: PMCPMC3987824.

11. Massey J. Causality, feedback and directed information. In: Proc Int Symp Information Theory Application (ISITA 1990). 1990:303-5.

12. Schreiber T. Measuring information transfer. Physical review letters. 2000;85(2):461-4. doi: 10.1103/PhysRevLett.85.461. PubMed PMID: 10991308.

13. Ince RA, Giordano BL, Kayser C, Rousselet GA, Gross J, Schyns PG. A statistical framework for neuroimaging data analysis based on mutual information estimated via a gaussian copula. Hum Brain Mapp. 2017;38(3):1541-73. doi: 10.1002/hbm.23471. PubMed PMID: 27860095.

14. Wibral M, Lizier JT, Priesemann V. Bits from Biology for Computational Intelligence. arXiv:14120291v1 [q-bioNC]. 2014.

15. Talairach J, Tournoux P. Co-Planar Stereotaxic Atlas of the Human Brain. NY: Thieme Medical Publishers; 1988.

16. Hickok G, Poeppel D. The cortical organization of speech processing. Nature reviews Neuroscience. 2007;8(5):393-402. doi: 10.1038/nrn2113. PubMed PMID: 17431404.
